# DNA-PK controls Apollo’s access to leading-end telomeres

**DOI:** 10.1093/nar/gkae105

**Published:** 2024-02-26

**Authors:** Ceylan Sonmez, Beatrice Toia, Patrik Eickhoff, Andreea Medeea Matei, Michael El Beyrouthy, Björn Wallner, Max E Douglas, Titia de Lange, Francisca Lottersberger

**Affiliations:** Department of Biomedical and Clinical Sciences, Linköping University, Linköping 58 183, Sweden; Department of Biomedical and Clinical Sciences, Linköping University, Linköping 58 183, Sweden; Chester Beatty Laboratories, The Institute of Cancer Research, 237 Fulham Road, London SW3 6JB, UK; Department of Biomedical and Clinical Sciences, Linköping University, Linköping 58 183, Sweden; Department of Biomedical and Clinical Sciences, Linköping University, Linköping 58 183, Sweden; Department of Physics, Chemistry and Biology, Linköping University, Linköping 58 183, Sweden; Chester Beatty Laboratories, The Institute of Cancer Research, 237 Fulham Road, London SW3 6JB, UK; Laboratory for Cell Biology and Genetics, The Rockefeller University, 1230 York Avenue, NY, NY 10021, USA; Department of Biomedical and Clinical Sciences, Linköping University, Linköping 58 183, Sweden

## Abstract

The complex formed by Ku70/80 and DNA-PKcs (DNA-PK) promotes the synapsis and the joining of double strand breaks (DSBs) during canonical non-homologous end joining (c-NHEJ). In c-NHEJ during V(D)J recombination, DNA-PK promotes the processing of the ends and the opening of the DNA hairpins by recruiting and/or activating the nuclease Artemis/DCLRE1C/SNM1C. Paradoxically, DNA-PK is also required to prevent the fusions of newly replicated leading-end telomeres. Here, we describe the role for DNA-PK in controlling Apollo/DCLRE1B/SNM1B, the nuclease that resects leading-end telomeres. We show that the telomeric function of Apollo requires DNA-PKcs’s kinase activity and the binding of Apollo to DNA-PK. Furthermore, AlphaFold-Multimer predicts that Apollo’s nuclease domain has extensive additional interactions with DNA-PKcs, and comparison to the cryo-EM structure of Artemis bound to DNA-PK phosphorylated on the ABCDE/Thr2609 cluster suggests that DNA-PK can similarly grant Apollo access to the DNA end. In agreement, the telomeric function of DNA-PK requires the ABCDE/Thr2609 cluster. These data reveal that resection of leading-end telomeres is regulated by DNA-PK through its binding to Apollo and its (auto)phosphorylation-dependent positioning of Apollo at the DNA end, analogous but not identical to DNA-PK dependent regulation of Artemis at hairpins.

## Graphical Abstract

**Graphical Abstract:**
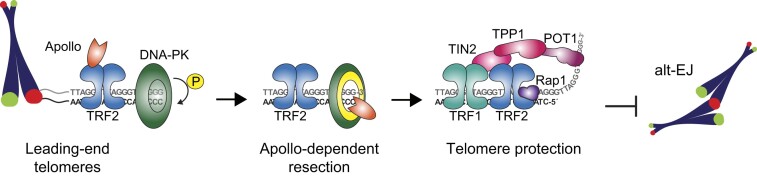

## Introduction

DNA-PK comprises a 470-kD kinase subunit (DNA-PKcs) and the heterodimeric Ku70/Ku80 DNA-end binding factor. DNA-PK’s primary function is to mediate canonical non-homologous end joining (c-NHEJ) of double strand breaks (DSBs) by recruiting DNA ligase IV (Lig4), its partner X-ray repair cross complementing 4 (XRCC4), and XRCC4-like factor (XLF) to DNA ends (reviewed in (1,2)). Recent biochemical, single-molecule and cryo-EM structural studies have revealed key mechanistic details of c-NHEJ (3–6). Following loading of Ku70/80 onto the two DNA ends, one copy of DNA-PKcs binds to each Ku heterodimer and promotes the synapsis of the ends into what is termed the XLF-mediated long-range (LR) synaptic complex. This LR complex stabilizes the association of Lig4/XRCC4 and XLF with DNA-PK, but the DNA ends are inaccessible and not aligned, requiring the transition to the short-range (SR) complex for ligation to take place. Although the details still need to be elucidated, the transition from XLF-mediated LR complex to SR complex is initiated by the autophosphorylation in*trans* of DNA-PKcs on a cluster of S/T-Q sites called the ABCDE/Thr2609 cluster, which induces the conformational changes required for the release of the kinase from the DNA ends and subsequent ligation of the two strands (3,5,7–10). The autophosphorylation *in trans* of a second cluster of S/T, the PQR/Ser2056 cluster, has also been shown to occur in the XLF-mediated LR complex to limit end processing (3,5,7,8).

Another LR synaptic complex, the Ku-mediated domain-swap dimer, is instead formed by the interaction *in trans* between Ku80 and DNA-PKcs binding the two opposite DNA ends (4). This complex is essential for the processing of DNA ends that are not directly ligatable, such as closed hairpins formed during V(D)J recombination by RAG recombinase. Such hairpins are opened by Artemis, an endonuclease/5′-exonuclease regulated by DNA-PK (11–15). How DNA-PK promotes Artemis’s access to the DNA ends and stimulates its nuclease activity is still an object of investigation. However, recent studies suggest that, first, a short (50 residues) region in the unstructured C-terminal tail of Artemis binds the FAT domain of DNA-PK (7,16,17). In this conformation, Artemis’s catalytic domain may face outwards, with no access to the DNA. After phosphorylation of the ABCDE/Thr2609 cluster either by DNA-PKcs itself (in *cis* or in *trans*) or by the DNA damage kinase ATM, DNA-PK undergoes a conformational change allowing the Artemis nuclease domains to access the DNA ends inside the DNA-binding groove of DNA-PK and to cleave them (7,15,17–20). DNA-PK then promotes the ligation of the ends after the transitions to the XLF-mediated LR and, subsequently, to the SR complex. However, since Artemis blocks DNA-PK in a self-inhibited conformation (7), the molecular mechanisms underlying DNA-PK (re)activation are still not clear. Furthermore, other end-processing factors, such as the Werner syndrome RecQ helicase (WRN) and the Mre11/Rad50/Nbs1 (MRN) complex, can also be activated by DNA-PKcs, but the underlying mechanism is less clear (21,22).

These distinct and context-specific roles of DNA-PK at DNA ends depend on multiple layers of regulation: the presence of both Ku70/80 and DNA-PKcs are required for end protection; DNA-PKcs autophosphorylation on the PQR/Ser2056 cluster is needed for c-NHEJ; and DNA-PKcs (auto)phosphorylation on ABDCE/Thr2609 cluster is necessary for Artemis recruitment and hairpin-opening as well as for the release of DNA-PK from the ends and ligation. This functional complexity could explain the differences observed in c-NHEJ and V(D)J recombination when DNA-PK is absent or inhibited (18,20,23–26).

The joining of telomeres through c-NHEJ is repressed by TRF2 (Telomeric repeat-binding factor 2), one of the subunits of the telomeric shelterin complex (27). Deletion of TRF2 results in extensive telomere–telomere fusions mediated by Ku70/80 and Lig4 (28–33). TRF2 is thought to block c-NHEJ by promoting the formation of t-loops, which hide the end of the chromosomes (34–36), as well as through other mechanisms (37–39). Essential for the protection of telomeres is the single-stranded G-rich 3′-overhang which invades the telomeric duplex to form the t-loop. While the 3′-overhang is thought to be a natural replication product at lagging-end telomeres, the newly synthesized leading-end telomeres should have (near) blunt ends and need to be processed. Formation of a 3′ overhang at leading-end telomeres is mediated by the 5′ nuclease activity of Apollo, which is brought to telomeres in S phase by TRF2 (40–47). Defects in Apollo recruitment and/or nuclease activity cause activation of ATM, reduction in the leading-end telomeric overhang and leading-end telomere fusions mediated by alternative EJ (alt-EJ) (42,44,46–50). In G1, Nbs1 competes for Apollo binding to TRF2, whereas in G2, hyper-resection by Apollo is counteracted by POT1 once a short overhang has been formed (51,52). However, it is not known if TRF2 recruitment alone is sufficient for Apollo to act right after replication and/or if there are additional layers of regulation.

Paradoxically for a factor involved in the re-joining of broken DNA ends, DNA-PK is present at telomeres, but its telomeric function has remained enigmatic (53,54). On one hand, DNA-PK deletion does not cause overt telomere deprotection except for occasional telomere fusions (55–61). On the other hand, chemical inhibition of DNA-PKcs causes fusions of newly replicated leading-end telomeres (55,62,63), and cells expressing a DNA-PKcs mutant lacking three phosphorylation sites in the ABCDE/Thr2609 cluster showed activation of the DNA damage response at leading-end telomeres in mitosis and a general increase in telomere fusion events (64).

Here, we demonstrate that kinase activity of DNA-PK and Apollo act in the same pathway to promote 3′-overhang formation and protection of leading-end telomeres from alt-EJ-mediated fusions. Accumulation of Apollo at telomeres depends solely on TRF2 and does not require DNA-PK or its kinase activity. However, we identify a domain in Apollo (amino acids 344–360) that promotes Apollo’s interaction with DNA-PK and is required for Apollo function at telomeres. This DNA-PK binding motif is similar to that of Artemis. Furthermore, DNA-PK activity and phosphorylation of DNA-PK on the ABCDE/Thr2609 cluster is required for Apollo’s function at telomeres. Alpha-Fold multimer version AFsample predicts that Apollo has extensive interactions with DNA-PK that would position the nuclease active site at the DNA end in a manner that depends on phosphorylation on the ABCDE/Thr2609 cluster. Therefore, we propose that DNA-PK activation at telomeres is required to grant Apollo access to the newly replicated leading-end telomeres in a timely manner.

## Material and methods

### Cell lines and cell culture

SV40-LT Apollo^F/F^ DNA-PKcs^+/+^, Apollo^F/F^ DNA-PKcs^−/−^, Apollo^F/F^ Ku70^−/−^, Apollo^F/F^ DNA-PKcs^−/−^ Ku70^−/−^, Apollo^F/F^ Lig4^−/−^, TRF2^F/F^ Lig4^−/−^, TRF2^F/F^ H2AX^−/−^, TRF2^F/−^ ATM^+/−^ and TRF2^F/−^ ATM^−/−^ MEFs have been previously described (30,46,50,65). DNA-PKcs^KD/KD^ and its controls DNA-PKcs^+/+^ and DNA-PKcs^−/−^ cell lines were a kind gift from Dr Shan Zha (20). DNA-PKcs^3A/3A^ and its control DNA-PKcs^+/+^ cell lines were a kind gift from Dr Benjamin Chen (66). All mouse embryonic fibroblasts (MEFs) were immortalized with pBabe-simian virus 40 large T antigen (SV40LT) vector and cultured in Dulbecco’s Modified Eagle Medium (DMEM, Cytiva) supplemented with 15% fetal bovine serum (FBS, Gibco), 1% non-essential amino acids (NEAAs, Cytiva), 1% L-glutamine (Cytiva), 1% penicillin/streptomycin (Cytiva) and 50 μM β-mercaptoethanol (Sigma). HT1080, 293T and Phoenix eco cells (ATCC, Rockville, MD) were cultured in DMEM supplemented with 10% HyClone Bovine Calf Serum (BCS, Cytiva), 1% NEAAs, 1% L-glutamine and 1% penicillin/streptomycin. DNA-PK inhibitors (DNA-PKi) NU7441 (KU-57788, Cayman), IC86621 (260962, Merck) and KU7026 (260961, Merck) were dissolved in dimethyl sulfoxide (DMSO) and added to the cells at a final concentration of 1.25–12.5 μM, 25–100 μM or 5–40 μM for 24 h, respectively. Bortezomib (PS-341, S1013, Selleckchem) was dissolved in ethanol and added to the cells at a final concentration of 100 nM for 6 h before harvest. ATM inhibitor (Ku-55933, Cayman) was dissolved in DMSO and added to the cells at a final concentration of 2.5 μM.

### Viral gene delivery

For retro- or lentiviral transduction, a total of 20 μg of plasmid DNA was transfected into Phoenix eco or 293T cells, respectively, using CaPO_4_ precipitation. The viral supernatant was filtered through a 0.45-μm filter, supplemented with 4 μg/ml polybrene, and used for the transduction of target cells. Cre-mediated deletions were achieved with three infections/day (6–12 h intervals) over 2 days with pMMP Hit & Run Cre retrovirus produced in Phoenix eco cells. Time-point 0 was set 12 h after the first Hit & Run Cre infection. For stable expression of HA-Apollo, six infections at 6–12 h intervals were performed before selection for 2–4 days in 2–4 μM Puromycin before Hit & Run Cre transduction.

Lentiviral particles containing the sgRNA against mouse Apollo (GTGATGGGAGAGCAGTAGAG) or human Apollo (CTGGTTCCAACGCAGCATGT) in lentiCRISPR v2 (Addgene plasmid # 52961, a gift from Feng Zhang) were produced in 293T cells and introduced into target MEFs/HT1080 cells with three infections/day (6–12 h intervals) over 1 or 2 days, followed by 2–3 days in 2–4 μM Puromycin before harvest.

Lentiviral particles containing the shRNAs for Ligase 3 (target: CCAGACTTCAAACGTCTCAAA; TRCN0000070978, Sigma) or DNA polymerase theta (PolQ; target: CGGTCCAACAAGGAAGGATTT; TRCN0000120312, Sigma) in a pLKO.1 vector (Openbiosystem) were produced in 293T cells and introduced into target MEFs with three infections/day (6–12 h intervals) over two days and selected for 3 days in 2–4 μM Puromycin before harvest.

### Chromosome orientation fluorescence *in situ* hybridization (CO-FISH) and immunofluorescence combined with fluorescent *in situ* hybridization (IF-FISH)

CO-FISH and IF-FISH were performed as previously described (29), with minor changes. Briefly, for CO-FISH, cells were treated with 10 μM BrdU:BrdC for 16 h and treated with 0.2 μg/ml Colcemid (Biowest/Roche) in the last 1–2 h before collection by trypsinization. Harvested cells were swollen in a hypotonic solution of 0.055–0.075 M KCl at 37°C for 15–30 min before fixation in methanol/acetic acid (3:1) overnight at 4°C. Cells were dropped onto glass slides and allowed to age overnight. Slides were then rehydrated in 1× PBS for 5 min, treated with 0.5 mg/ml RNase A (R5000; Sigma) in PBS for 10 min at 37°C, stained with 0.5 μg/ml Hoechst 33258 (B2883; Sigma) in 2× saline-sodium citrate (SSC) for 15 min, and exposed to 5.4 × 10^3^J/m^2^ 365-nm UV light (Stratalinker 1800 UV irradiator), followed by digestion of the BrdU/BrdC substituted DNA strand with 800 U of 10 U/μl Exonuclease III (M1815, Promega) for 30 min. The slides were then dehydrated through an ethanol series of 70%, 95% and 100% and allowed to air-dry. Telomere ends were hybridized with Alexa Fluor 488-OO-(TTAGGG)_3_ or Cy3-OO-(TTAGGG)_3_ in hybridization solution (70% formamide, 1 mg/ml blocking reagent [1109617601, Roche], and 10 mM Tris-HCl pH 7.2) for 2 h followed by a few seconds wash in 70% formamide/10 mM Tris-HCl, pH 7.2. The slides were then hybridized with Alexa Fluor 647-OO-(CCCTAA)_3_ or Cy3-OO-(CCCTAA)_3_ (PNA Bio) in hybridization solution for 2 h. Slides were washed twice in 70% formamide; 0.1% Bovine Serum Albumin (BSA); 10 mM Tris-HCl, pH 7.2 for 15 min each, and thrice in 0.08% Tween-20, 0.15 M NaCl, 0.1 M Tris-HCl, pH 7.2 or PBS for 5 min each. Chromosomal DNA was counterstained with the addition of 4′,6-diamidino-2-phenylindole (DAPI) (D1306, Invitrogen) to the second wash. Slides were left to air-dry and mounted in antifade reagent (Prolong Gold Antifade P36934, Fisher). For HT1080, the same protocol was followed with the following modifications: slides were exposed for 10 min to a total of 2.4 × 10^4^ J/m^2^ 365-nm UV light; the staining was performed in hybridization solution with Alexa Fluor 647-OO-(TTAGGG)_3_ and Cy3-OO-(CCCTAA)_3_ (PNA Bio).

For IF-FISH, MEFs were cultured on poly-D-Lysine (A3890401, Gibco) pre-coated coverslips for 1–2 days. Cells were rinsed in cold PBS and pre-extracted using cold Triton X-100 buffer (0.1% Triton X-100; 20 mM Hepes-KOH, pH 7.9; 50 mM NaCl; 3 mM MgCl_2_; 300 mM sucrose) for 20 min on ice, followed by two washes in 1× PBS at RT, before fixation for 10 min at RT with 3% paraformaldehyde/2% sucrose. Cells were re-permeabilized for 15 min with 0.1% Triton X-100 buffer before blocking and staining in Blocking solution (1 mg/ml BSA; 3% goat serum; 0.1% Triton X-100; 1mM EDTA, pH 8, in PBS). HA (HA.11, #901502, Biolegend) or γ-H2AX (JBW301, Millipore) primary antibodies, and secondary anti-mouse Alexa Fluor 647 antibody (A32728, Invitrogen), were incubated overnight at 4°C or 1–2 h at RT, respectively. Samples were fixed again in 3% paraformaldehyde/2% sucrose for 10 min at RT before dehydration through an ethanol series of 70%, 95% and 100%, and allowed to air-dry. Hybridization was performed with Alexa Fluor 488-OO-(TTAGGG)_3_ in hybridization solution (70% formamide; 0.5% blocking reagent (1109617601, Sigma); 10 mM Tris-HCl, pH 7.2) for 10 min at 45°C on a heat block, followed by incubation at RT for 2 h. After two washes in washing solution (70% formamide; 10 mM Tris-HCl, pH 7.2), and three in PBS, where DAPI was added to the second wash to stain the cell nuclei, coverslips were left to air-dry and mounted in antifade reagent.

HT1080 pictures were acquired on Leica DMi8 with a 63×/1.2 objective using LAS X software. 2D-maximum intensity projection images were obtained using Fiji software (67). All other pictures were acquired on a DeltaVision RT microscope system (Applied Precision) with a PlanApo 60 × 1.40 NA objective lens (Olympus America, Inc.) at 1 × 1 binning and multiple 0.2 μm Z-stacks using SoftWoRx software. Images were deconvolved, and 2D-maximum intensity projection images were obtained using SoftWoRx software. Chromatid and chromosome-type fusions were analyzed using Fiji software (67) after arbitrary assignment of red for both (TTAGGG) _3_-probes and green for both (CCCTAA)_3_-probes. Semi-automated analysis and quantification of colocalization was performed using CellProfiler (68) with the following pipeline: image cropping to remove edge artifacts due to deconvolution; channel intensity rescaling to cover the full histogram range value; ‘speckle features enhancement’ with a size cut-off of 5 pixels to increase detection sensitivity and remove background/artifact aggregates; ‘channel-wise primary objects identification’ to detect individual nuclei (diameter range: 50–1000 pixels, threshold correction: 1.5) and individual foci (diameter range: 2–15 pixels, threshold correction: 1–4); correlation of the foci coordinates in the different channels and with the respective nuclei to define colocalization events. Nuclei with <10 or 20 detected PNA foci were discarded for HA-Apollo and γ-H2AX analysis, respectively.

### Immunoblotting

Cells were lysed in 2× Laemmli buffer at 5 × 10^3^ or 1 × 10^4^ cells/μl and the lysate was denatured for 10 min at 95°C before shearing with an insulin needle or sonication at 40% amplitude for 15 s, 5 s ON and 5 s OFF (Fisherbrand sonicator, Model; FB705; power 700 W; 2000 Park Lane, Pittsburgh, PA, 15275). Lysate equivalent to 1–2 × 10^5^ cells was resolved using SDS/PAGE and transferred to a nitrocellulose membrane. Western blot was performed with 5% milk in PBS containing 0.1% (v/v) Tween-20 (PBS-T) using the following antibodies: β-actin (#3700; Cell Signaling), Chk2 (BD 611570, BD Biosciences), DCLRE1B/Apollo (HPA064934, Atlas Antibodies), DNA-PKcs (SC-1552, SC-5282, SC-390495 and SC-390849; Santa Cruz Biotechnology), HA (HA.11, #901502 Biolegend), Ku70 (sc-17789 or sc-1487, Santa Cruz Biotechnology), Myc (9B11, mAb 2276; Cell Signaling), TRF2 (#13136, Cell Signaling) and secondary anti-Mouse/anti-Rabbit IgG HRP (Cytiva). Signals were detected according to the manufacturer’s instructions using chemiluminescence western blotting detection reagents (Cytiva) on either BioMax MR film (Kodak) or ChemiDoc (Bio-Rad) imaging systems.

### In-gel analysis of single-stranded telomeric DNA

Telomeric DNA was analyzed on Clamped homogenous electric field (CHEF) gels as previously described (29). Briefly, cells were harvested by trypsinization, resuspended in PBS, mixed with 2% agarose (1:1 ratio) at 50°C and cast in a plug mold 0.7–1 × 10^6^ cells/plug. Plugs were digested overnight at 50°C in 1 mg/ml proteinase K (03115879001; Roche) in digest buffer (100 mM EDTA, 0.2% sodium deoxycholate, and 1% sodium lauryl sarcosine) and washed five times in TE. After DNA digestion overnight at 37°C by 60U MboI (#R0147; New England BioLabs), plugs were then washed in 0.5× TBE twice before loading on a 1% agarose/0.5×TBE gel. DNA was resolved by a CHEF-DRII PFGE apparatus (Bio-Rad) for 20 h, with the following settings: initial pulse, 5 s; final pulse, 5 s; 6 V/cm at 14°C. Gel was dried and hybridized overnight at 50°C with γ-^32^P-ATP end-labeled TelC (AACCCT)_4_ probe in Church mix (0.5 M sodium phosphate buffer pH 7.2, 1 mM EDTA, 7% SDS, 1% BSA). After three washes in 4× SSC and one in 4× SSC/0.1% SDS at 55°C, the gel was exposed for one/two days before acquisition of the single-stranded telomere signal by PhosphoImager. The gel was then denatured with 1.5 M NaCl/0.5 M NaOH for one hour, neutralized with two washes of 1 h each in 0.5 M Tris-HCl pH 7.0/3 M NaCl, pre-hybridized for 30 min at 55°C in Church mix, and hybridized overnight at 55°C before being washed and exposed as described before for the acquisition of the total telomere signal. Quantification of the signals in each lane was done using ImageQuant software. After subtraction of the background, the single-stranded signal was normalized to the total telomeric DNA signal in the same lane. The indicated control value was set to 1, and all the other values were given as a percentage of this value.

### Generation of HA-Apollo mutant alleles

PCR-mediated site-directed mutagenesis was used to generate pLPC retroviral vector expressing FLAG-[HA]2 tagged mouse Apollo-AA or Apollo-DPK mutant alleles using previously published construct (46) as templates and the following primers:

Apollo^T162A^-Fw: GGTTCTTCCTTCCCGACAAGAGGCTGCTCAGCAGATTGTCCAGCTAATCCGAC and

Apollo^T162A^-Rv: GTCGGATTAGCTGGACAATCTGCTGAGCAGCCTCTTGTCGGGAAGGAAGAACC.

Apollo^T350A^-Fw: AGAAAGGAGGCTCAAGAGGCCAAGAGCTCAGGGTGTTGTGTTTGAATCCCCTG and

Apollo^T350A^-Rv: CAGGGGATTCAAACACAACACCCTGAGCTCTTGGCCTCTTGAGCCTCCTTTCT.

Apollo^ΔDNA-PK^-Fw: AAAGCTAATCAGGTTAAAGTTGAC and

Apollo^ΔDNA-PK^-Rv: CCTTTCTAACTGCCAAAGCACATT

### Coimmunoprecipitation

For Myc-TRF2 pulldown with HA-Apollo, 293T cells were plated in 10-cm dishes 24 h prior to transfection by CaPO_4_ precipitation using 10 μg of each indicated plasmid. The medium was changed 12 h after transfection with fresh medium containing DMSO or 12.5 μM NU7441 for 24 h. Cells were harvested using a cell scraper in cold PBS at 4°C, collected by centrifugation, and resuspended in 0.5 ml hypotonic lysis buffer (50 mM Tris-HCl pH 7.4, 1% Triton-X-100, 0.1% SDS, 150 mM NaCl, 1 mM EDTA, 1 mM dithiothreitol (DTT), 1 mM phenylmethylsulfonyl fluoride (PMSF)), complete protease inhibitor mix (#11836170001, Roche) and phosphatase inhibitor (#4906845001, Roche). NaCl concentration was raised to 400 mM for 5 min on ice and then lowered to 200 mM to extract nuclear proteins. After centrifugation at maximum speed for 10 min at 4°C, samples were incubated at 4°C overnight with anti-HA (HA.11; 901502 Biolegend) antibody on a nutator. After incubation with pre-blocked protein-G Sepharose beads for one hour, pellets were washed 4× with lysis buffer without detergents. Immunoprecipitated proteins were eluted with 60 μl of 2× Laemmli buffer and boiled for 5 min before separation by SDS-PAGE.

For DNA-PK pulldown with HA-Apollo, 20 μg of plasmid was used to transfect 293T cells plated in 10 cm dishes 24 h prior to transfection by CaPO_4_ precipitation. Cells were harvested 40 h after transfection as previously described. Co-IP was performed as previously described for Artemis with minor modifications (14). All steps were performed at 4°C. Briefly, cells were lysed in 0.5 ml lysis buffer (25 mM HEPES pH 7.4, 150 mM KCl, 10 mM MgCl_2_, 10% glycerol, 2 mM DTT, 1 mM EDTA, 8 mM β-mercaptoethanol) supplemented with complete protease inhibitor mix and phosphatase inhibitor and kept 10 min on ice before sonication at 40% amplitude for 30 s; 5 s ON and 5 s OFF. After centrifugation at maximum speed for 30 min, the lysate was pre-cleared with protein-G Sepharose beads in lysis buffer (protein-G Sepharose 4 fast flow; 17061801 Cytiva) for 1 h followed by overnight incubation with anti-HA antibody at 4°C on an end-to-end nutator. Samples were then incubated with protein-G Sepharose beads pre-blocked in 0.2%BSA/PBS for 4 h at 4°C on an end-to-end nutator and washed 4 × 5 min with lysis buffer on an end-to-end nutator. The immunoprecipitated proteins were then eluted with 40 μl of 2× Laemmli buffer and boiled for 5 min before separation by SDS-PAGE.

### Structural modeling

AFsample (69), an improved version of AlphaFold-Multimer (70) deemed the best method in CASP15 (71) for multimer assembly, was used to predict the interaction of mouse Apollo with mouse DNA-PKcs amino acids 1981–2760. Multiple sequence alignments (MSAs) were constructed using the –reduce_db option, resulting in 5666 and 1070 homologous sequences in the Apollo and DNA-PKcs, MSAs, respectively. Templates to known PDB structures were not used to avoid any bias to different conformational states and experimental conditions. In total, 816 models of Apollo:DNA-PKcs were generated using *multimer_v1* neural network weights with –dropout enabled at inference to get more diversity in the pool of sampled models. The model with the highest reported ranking confidence was relaxed in the Amber99sb force field (72) using openMM (73), and used as a representative model of the interaction.

### Sequence alignments

Apollo, Artemis and DNA-PKcs sequences were obtained from Uniprot (https://www.uniprot.org). Sequences were aligned in SnapGene (GSL Biotech) using Clustal Omega or MUSCLE multiple sequence comparison. Alignments were colored either compared to mouse reference sequence or with Zappo coloring (physico-chemical properties).

### Quantification and statistical analysis

Quantification and statistical analysis were performed GraphPad on three or more independent experiments, as indicated. For CO-FISH analysis, at least ten metaphases per condition were scored for each experiment. For IF-FISH analysis, >80 nuclei per condition per experiment were scored. For overhang analysis, the normalized overhang signal was expressed relative to the untreated control and independently for each cell line. Significance was assessed by calculating the p-value using non-parametric Kruskal–Wallis ANOVA for multiple comparisons or Mann–Whitney *t*-test for single cell analysis and one- or two-way ANOVA for multiple comparisons for population analysis. *P*-values ≤ 0.05 were considered statistically significant.

## Results

### DNA-PKcs kinase activity and Apollo act in the same pathway to prevent alt-EJ of leading-end telomeres

**Figure 1.**
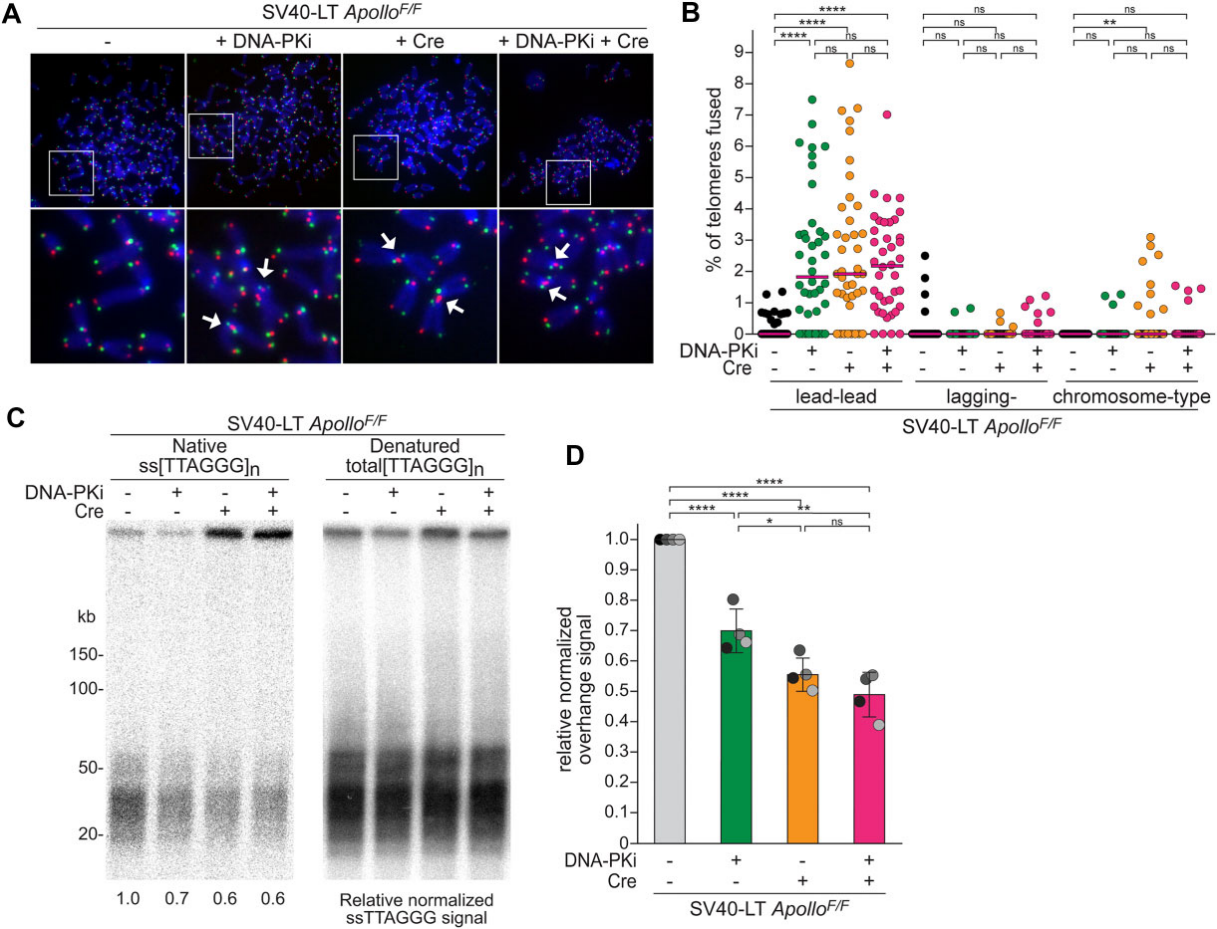
DNA-PK inhibition prevents Apollo-dependent leading-end telomere protection. (**A**) Representative chromosome orientation fluorescence *in situ* hybridization (CO-FISH) of metaphase spreads of *Apollo^F/F^* MEFs immortalized with SV40LT 96 h after Hit & Run Cre treatment and/or after 24 h treatment with DNA-PKcs inhibitor NU7441 (DNA-PKi). Leading and lagging-end telomeres were detected with Cy3-(TTAGGG)_3_ (red) and Alexa488-(CCCTAA)_3_ (green) probes, respectively. DNA was stained with DAPI (blue). Arrows indicate leading-end telomere fusions. (**B**) Quantification of telomere fusions as shown in (A). Each dot represents the percentage of telomeres fused in one metaphase, aggregated for chromatid-type (involving two leading-end telomeres or two lagging-end telomeres) or chromosome-type fusions. Bars represent the median of telomere fusions in 40 metaphases across *n* = 4 independent experiments (10 metaphases per experiment). (**C**) Telomeric overhang assay on *Apollo^F/F^* MEFs treated as in (A). Genomic DNA was digested with MboI, and the single-stranded telomeric DNA was detected in-gel with end-labeled ^32^P-(AACCCT)_4_ (native, left panel). DNA was then denatured, and the gel was rehybridized with the same probe to determine the total telomeric signal (denatured, right panel). The ssTTAGGG signal was normalized to the total telomeric DNA in the same lane. The normalized no Cre value was set to 1 and all the other values were calculated relative to it. (**D**) Quantification of the relative overhang signal as detected in (C) for *n* = 4 independent experiments (indicated by different shades), with means and SDs. Statistical analysis from non-parametric Kruskal–Wallis ANOVA test for multiple comparisons (B) or ordinary one-way ANOVA for multiple comparisons (D), ****P< 0.0001, ***P< 0.001, **P< 0.01, *P< 0.05; n.s., not significant.

Previous reports showed that DNA-PK inhibitors IC86621 and NU7026 induce leading-end telomere fusions as does deletion of Apollo, and the severity of the telomere fusion phenotype is similar in these two settings (42,46,48,63,74,75). Therefore, we explored the possibility of an epistatic relationship between DNA-PK and Apollo. As expected, we observed leading-end telomere fusions at similar frequencies both in MEFs where Apollo was deleted with Cre, and MEFs treated with NU7441 (76), one of the most potent and selective DNA-PKcs inhibitor available (hereafter referred to as DNA-PKi) (Figure 1A,B). DNA-PKi did not exacerbate the leading-end telomere fusion phenotype of Apollo-deficient cells (Figure 1A,B), suggesting that DNA-PK and Apollo act in the same pathway to protect leading-end telomeres.

Importantly, DNA-PKi reduced the 3′-overhang signals to approximately the same extent as the deletion of Apollo, and the overhang phenotype of Apollo deletion was not exacerbated by DNA-PKi (Figure 1C,D). Furthermore, DNA-PKi induced a DNA damage response at telomeres similar to Apollo deletion, as evident from the appearance of γ-H2AX-marked telomere dysfunction-induced foci (TIFs), although it did not induce the phosphorylation of Chk2 (P-Chk2) (Supplementary Figure S1A–C). Finally, similar to our recent data on Apollo deletion (50), chromatid fusions induced by DNA-PKi were abolished in the absence of alt-EJ factors Ligase 3 (Lig3) and PolQ (Supplementary Figure S2A, B), but not of c-NHEJ Lig4 (Supplementary Figure S2C,D), pointing to alt-EJ as the predominant mechanism promoting telomere fusions in cells with inhibited DNA-PK activity. The reason for the lack of Chk2 phosphorylation upon DNA-PK inhibition is unclear, and we cannot explain the difference with prior reports showing that the other DNA-PKi NU7026, causes telomere fusions mostly dependent on c-NHEJ (74,75). Nevertheless, our data suggest that DNA-PK kinase activity is required for Apollo to form the 3′-overhang and to protect the leading-end telomeres from fusion.

Unexpectedly, we observed that DNA-PKi had an off-target effect on the stability of HA-tagged Apollo expressed through retroviral transduction (Supplementary Figure S3). DNA-PKi strongly reduced the levels of HA-Apollo in a time and dose-dependent manner (Supplementary Figure S3A,B). Remarkably, the effect of DNA-PKi was independent of the presence of DNA-PK. DNA-PKi-dependent degradation of HA-Apollo was comparable in MEFs expressing or lacking the DNA-PK components DNA-PKcs and/or Ku70, and in MEFs expressing the catalytically inactive DNA-PKcs-D3922A mutant (DNA-PKcs^KD/KD^) (20), (Supplementary Figure S3C,D). This effect could also be observed with other DNA-PK inhibitors (NU7026 and IC86621), even at low concentrations that do not trigger chromatid fusions (63,74,75) (Supplementary Figure S3E,F). The effect of DNA-PKi was attenuated by Bortezomib (Supplementary Figure S3G), indicating that it is, at least partially, due to proteasomal degradation. Since mouse endogenous Apollo could not be detected using commercial antibodies, we could not test whether DNA-PKi also affects the levels of endogenous Apollo.

**Figure 2.**
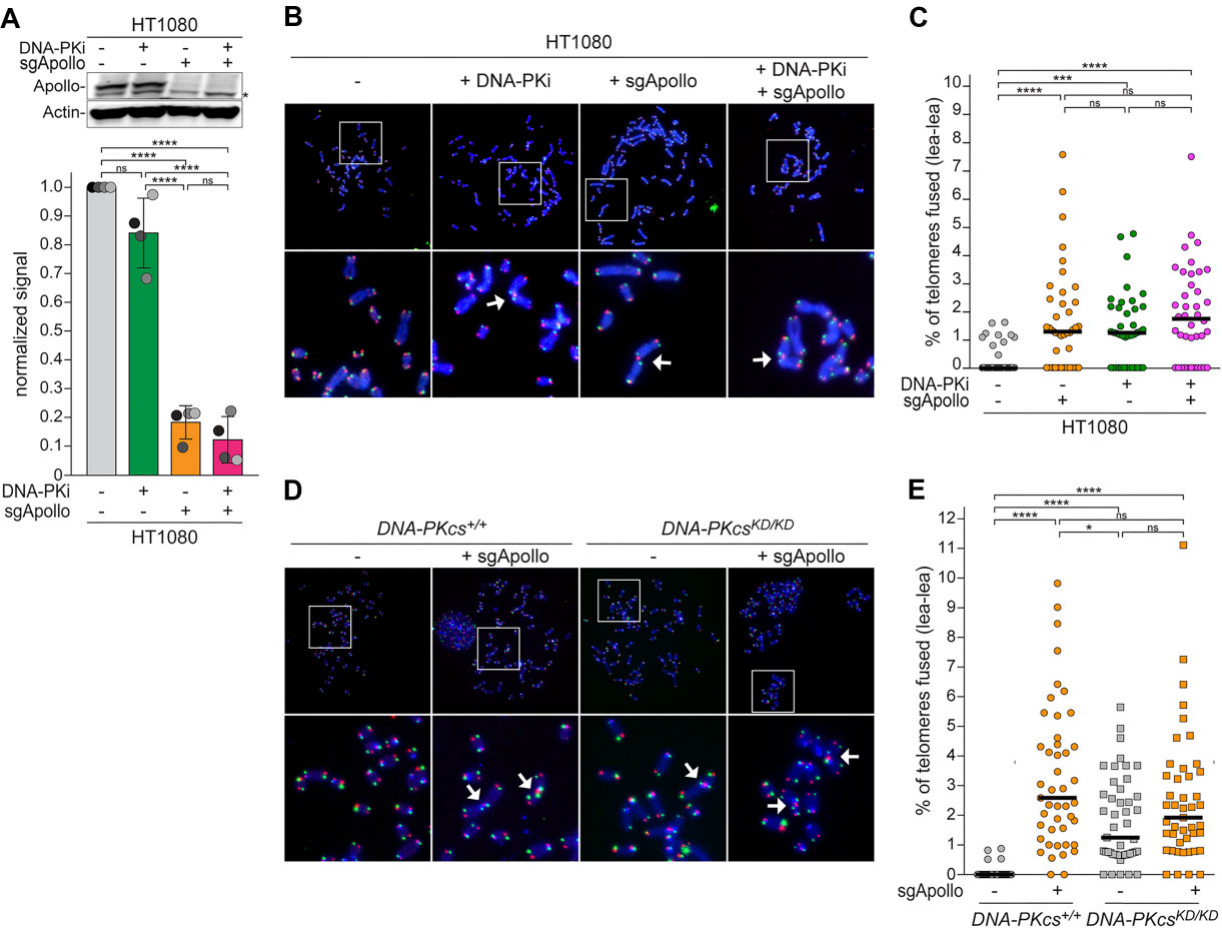
DNA-PK kinase activity promotes Apollo-dependent leading-end telomere protection. (**A**) Immunoblots for Apollo and actin loading control in HT1080 human cancer cells 120 h after lentiviral transduction with sgRNA targeting human Apollo and/or after 24 h treatment with DNA-PKi. Graph represents Apollo levels normalized over actin in *n* = 4 independent experiments, with mean and SD. (**B**) Representative CO-FISH of metaphase spreads of HT1080 cells treated as described in (A). Leading and lagging-end telomeres were detected with Alexa Fluor 647-OO-(TTAGGG)_3_ (red) and Cy3-OO-(CCCTAA)_3_ (green), respectively. DNA was stained with DAPI (blue). Arrows indicate leading-end telomere fusions. (**C**) Quantification of leading-end telomere fusions as described in (B). Each dot represents the percentage of leading-end telomere fusions in one metaphase. Bars represent the median of telomere fusions in 40 metaphases across *n* = 4 independent experiments (10 metaphases each). **(D** and **E)** CO-FISH metaphase analysis and quantification of leading-end telomere fusions as described in (B) and (C) for *DNA-PKcs^+/+^* or *DNA-PKcs^KD/KD^* MEFs 108 h after lentiviral transduction with an sgRNA targeting mouse Apollo or the empty vector control. Bars represent the median of telomere fusions in 45 metaphases across *n* = 3 independent experiments (15 metaphases per experiment). Statistical analysis by ordinary one-way ANOVA for multiple comparisons (A) or by non-parametric Kruskal–Wallis ANOVA test for multiple comparisons (C,E). Statistical significance was indicated by ****P < 0.0001, ***P < 0.001, **P < 0.01, *P < 0.05; n.s., not significant.

Given these off-target effects of DNA-PKi in mouse cells, we tested the effect of DNA-PK inhibition in the HT1080 human cancer cell line. In HT1080 cells, DNA-PKi caused only a minimal reduction in endogenous Apollo (Figure 2A). Nevertheless, DNA-PKi caused similar levels of leading-end telomere fusions as CRISPR/Cas9 deletion of Apollo (Figure 2A–C). Importantly, the effect of DNA-PKi and Apollo deletion were not additive (Figure 2C), arguing that the epistatic relationship between Apollo and DNA-PK kinase activity is not due to DNA-PKi inducing the degradation of the endogenous Apollo. Finally, leading-end telomere fusions were also observed in DNA-PKcs^KD/KD^ MEFs, independently of DNA-PKi treatment (Supplementary Figure 4A,B) or the presence of an sgRNA targeting mouse Apollo (Figure 2D,E). These data confirm that DNA-PK kinase activity is required for Apollo to form the 3′-overhang and to protect the leading-end telomeres from fusion.

### The target of DNA-PK kinase activity is DNA-PK, not Apollo's S/T-Q sites or recruitment mechanism

**Figure 3.**
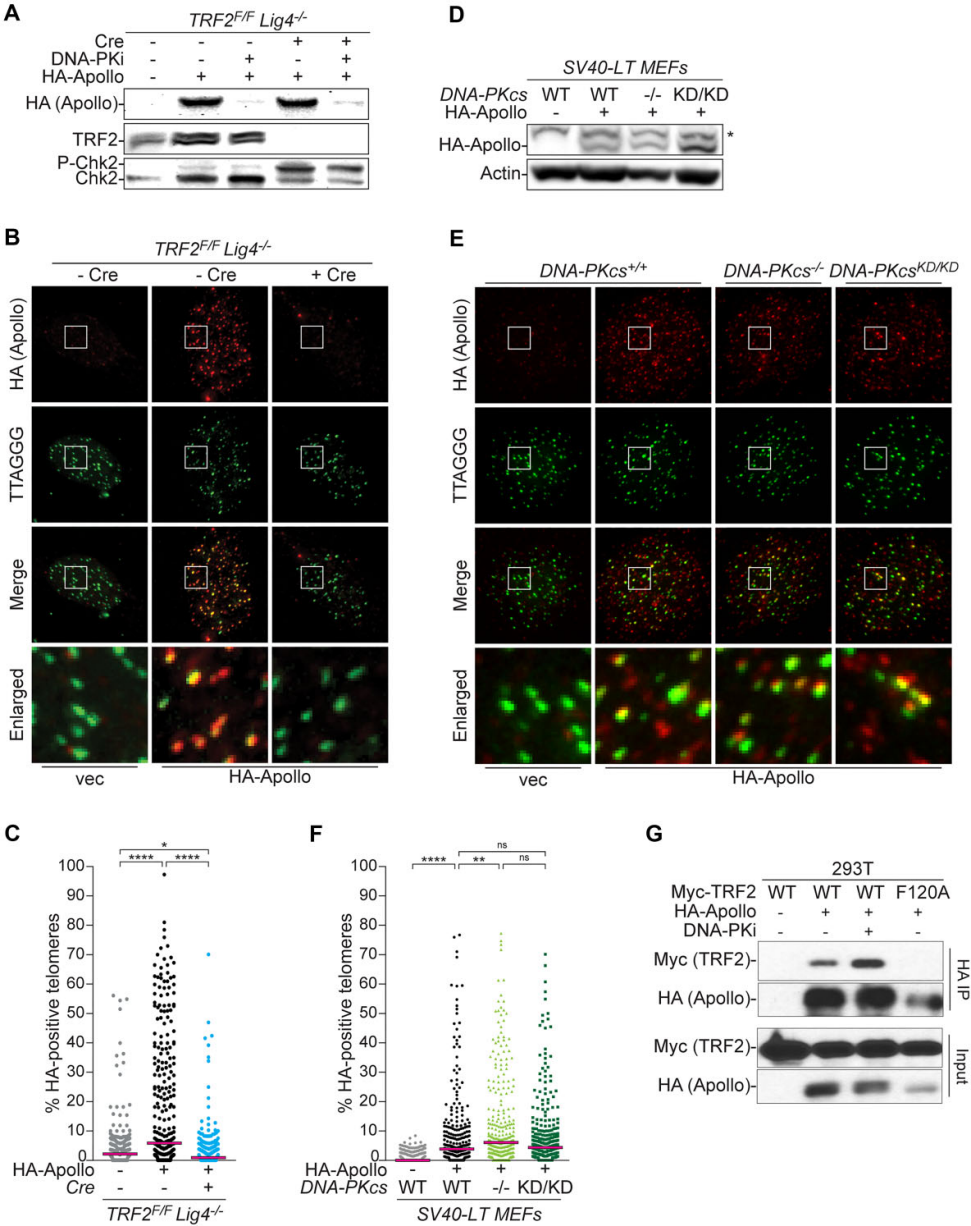
DNA-PK kinase activity is not required for Apollo recruitment to telomeres. (**A**) Immunoblots for HA-Apollo (anti-HA), TRF2 and phosphorylated Chk2 in SV40LT-immortalized *TRF2^F/F^ Lig4 ^-/−^* MEFs after retroviral transduction transduced with an empty vector (EV) or HA-Apollo, 108 h after Hit & Run Cre-mediated deletion of TRF2 and/or 24 h DNA-PKi treatment. **(B** and **C)** Representative immunofluorescence-fluorescence in-situ hybridization (IF-FISH) images and quantification of HA-Apollo localization at telomeres in the same MEFs as described in (A) 120 h after Hit & Run Cre transduction or without any treatment. Apollo and telomeres were detected using anti-HA (red) or Alexa488-OO-(TTAGGG)_3_ probe (green), respectively. Approximately 300 nuclei were analyzed for each condition over *n* = 3 independent experiments (100 nuclei per experiment). Median bar is indicated. (**D**) Immunoblots for HA-Apollo (anti-DCLRE1B, Atlas) and actin in SV40-LT-immortalized *DNA-PKcs^+/+^*, *DNA-PKcs^−/−^* or *DNA-PKcs^KD/KD^* MEFs transduced with an empty vector (EV) or HA-Apollo. * aspecific band. (**E** and **F**) IF-FISH analysis and quantification of HA-Apollo localization at telomeres in the SV40-LT-immortalized *DNA-PKcs^+/+^*, *DNA-PKcs^−/−^* or *DNA-PKcsK^KD/KD^* MEFs transduced with an empty vector (EV) or HA-Apollo, analyzed as described in (B, C). For HA-Apollo, 300 nuclei over *n* = 3 independent experiments are shown (100 nuclei per experiment), with median bar. As negative control, 296 nuclei (at least 90 nuclei per experiment) were analyzed in parallel for EV. (**G**) Co-immunoprecipitation (Co-IP) of Myc-mTRF2 (WT or F120A) with HA-mApollo in 293T cells without or after 24 h treatment with DNA-PKi. Pull down was performed with anti-HA antibody. HA IP (first and second panels) and Input (third and fourth panels) were analyzed with anti Myc (first and third panels) or HA (second and fourth panels) antibodies. MYC-mTRF2-F120A (F120A) was used as negative control. Statistical analysis by non-parametric Kruskal–Wallis ANOVA test for multiple comparisons (C, F). Statistical significance was indicated by ****P< 0.0001, ***P< 0.001, **P< 0.01, *P< 0.05; n.s., not significant.

A possible explanation for the epistatic relationship between Apollo and DNA-PK is that DNA-PK promotes Apollo localization at telomeres together with TRF2. We addressed this question by monitoring the localization of HA-Apollo to telomeres by immunofluorescence (IF). As expected, HA-Apollo was detected at telomeres in cells expressing TRF2 but not in TRF2^F/F^ Lig4^−/−^ MEFs after TRF2 deletion with Cre (Figure 3A–C). By contrast, the telomeric localization of HA-Apollo appeared unaffected in cells that either lacked DNA-PK or carried the kinase-dead allele of DNA-PKcs (Figure 3D–F). Consistent with DNA-PK not being required for the TRF2-mediated recruitment of Apollo to telomeres, co-immunoprecipitation (co-IP) experiments showed that the interaction of HA-Apollo and Myc-TRF2 was not affected by DNA-PKi (Figure 3G).

We also excluded that the effect of DNA-PKi involves the phosphorylation of H2AX or ATM, both known targets of DNA-PKcs kinase activity (77–79). In fact, DNA-PKi treatment in H2AX or ATM null cells resulted in the reduction of the 3′-overhang signal and the increase in leading-end telomere fusions similar to the control cells (Supplementary Figure S5A–D), consistent with previous reports (63,74).

**Figure 4.**
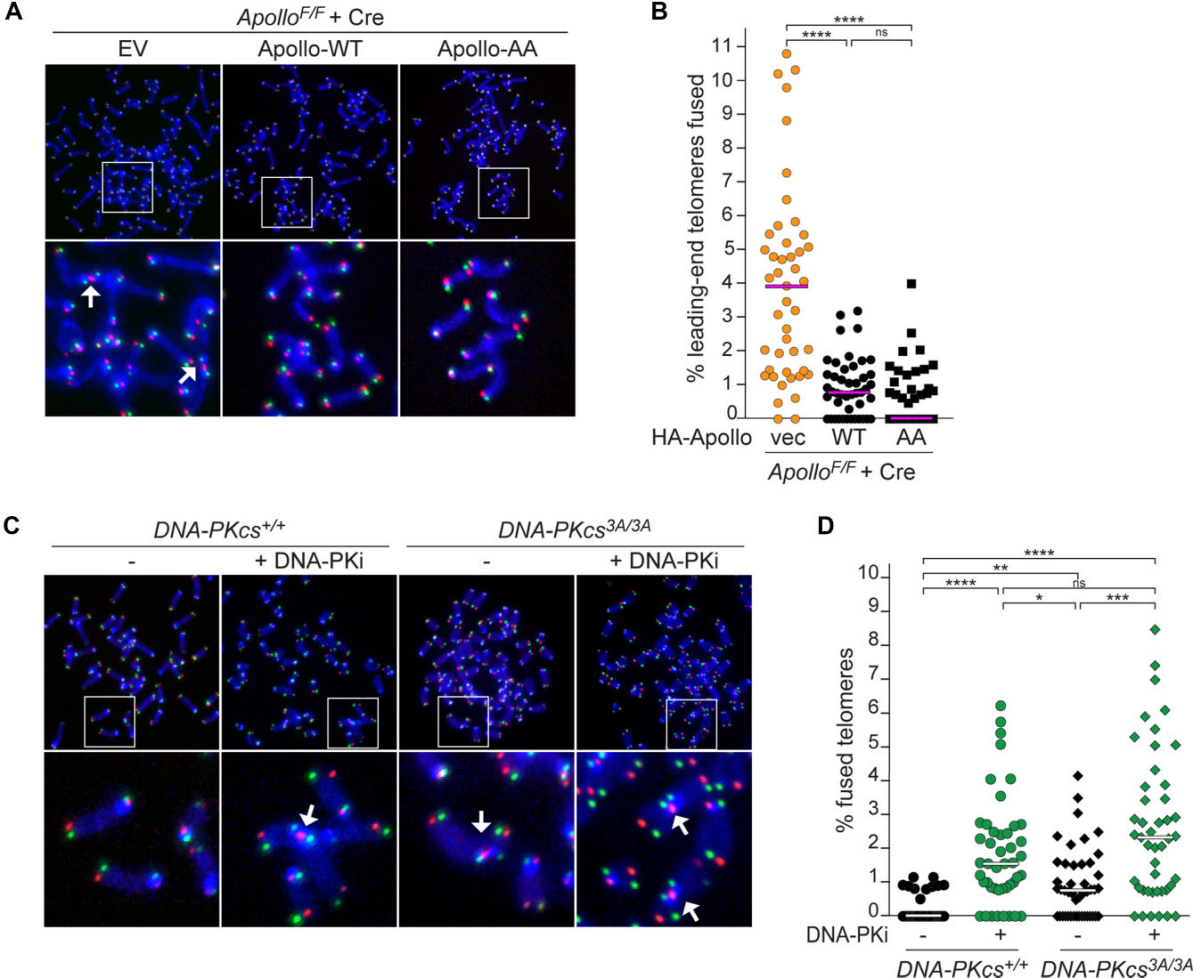
DNA-PK, not Apollo S/T-Q sites, is the target of DNA-PK kinase activity. **(A** and **B)** CO-FISH metaphase analysis and quantification of leading-end telomere fusions in SV40LT-immortalized *Apollo^F/F^* MEFs transduced with an empty vector (EV), HA-Apollo (WT) or HA-Apollo-AA (AA), 96 h after Hit & Run Cre-mediated deletion of Apollo. The graph represents 45 metaphases over *n* = 3 independent experiments, with median bars. **(C** and **D)** CO-FISH and quantification of leading-end telomere fusions in *DNA-PKcs^+/+^* or *DNA-PKcs^3A/3A^* MEFs with or without 24 h treatment with DNA-PKi for 45 metaphases over *n* = 3 independent experiments, with median bar. Statistical analysis by non-parametric Kruskal–Wallis ANOVA test for multiple comparisons (B, D). Statistical significance was indicated by ****P< 0.0001, ***P< 0.001, **P< 0.01, *P< 0.05; n.s., not significant.

Mouse Apollo contains two S/T-Q sites (T162 and T350) that are considered canonical targets of DNA-PK. Although they have never been shown to be phosphorylated and only one of them is conserved in most mammals (Supplementary Figure S6A), we investigated their role in telomere protection. However, an Apollo mutant where both threonine residues are mutated to alanines (Apollo-AA) was indistinguishable from wild-type Apollo (Apollo-WT) in its recruitment at telomeres (Supplementary Figure S6B–D), and in the maintenance of the 3′-overhang and the protection of telomeres from fusion (Figure 4A,B and Supplementary Figure S6E,F) when expressed in Cre-treated Apollo^F/F^. Therefore, S/T-Q phosphorylation of Apollo is not required for telomere end-processing.

Finally, we tested whether (auto)phosphorylation of DNA-PKcs is required for the telomeric function of DNA-PK, since MEFs carrying a DNA-PKcs mutant with three threonines out of the five phosphorylation sites in the mouse ABCDE/Thr2609 cluster changed to alanine (DNA-PK^3A/3A^) have high levels of telomere fusions (64). Indeed, DNA-PK^3A/3A^ MEFs confirmed the fusion phenotype, specifically involving leading-end telomeres (Figure 4C,D). DNA-PKi treatment increased the leading-end telomere fusion events in DNA-PK^3A/3A^ MEFs, but not more than what is observed in the DNA-PK^+/+^ control cells (Figure 4C,D). Although it is possible that other substrates of DNA-PK and/or other DNA-PKcs phosphorylation sites are involved, this result suggests that, in the context of telomere processing, the relevant target of DNA-PK kinase activity is the ABCDE/Thr2609 cluster of DNA-PKcs.

### Phosphorylation on the DNA-PK ABCDE/Thr2609 cluster is predicted to provide Apollo access to DNA ends

**Figure 5.**
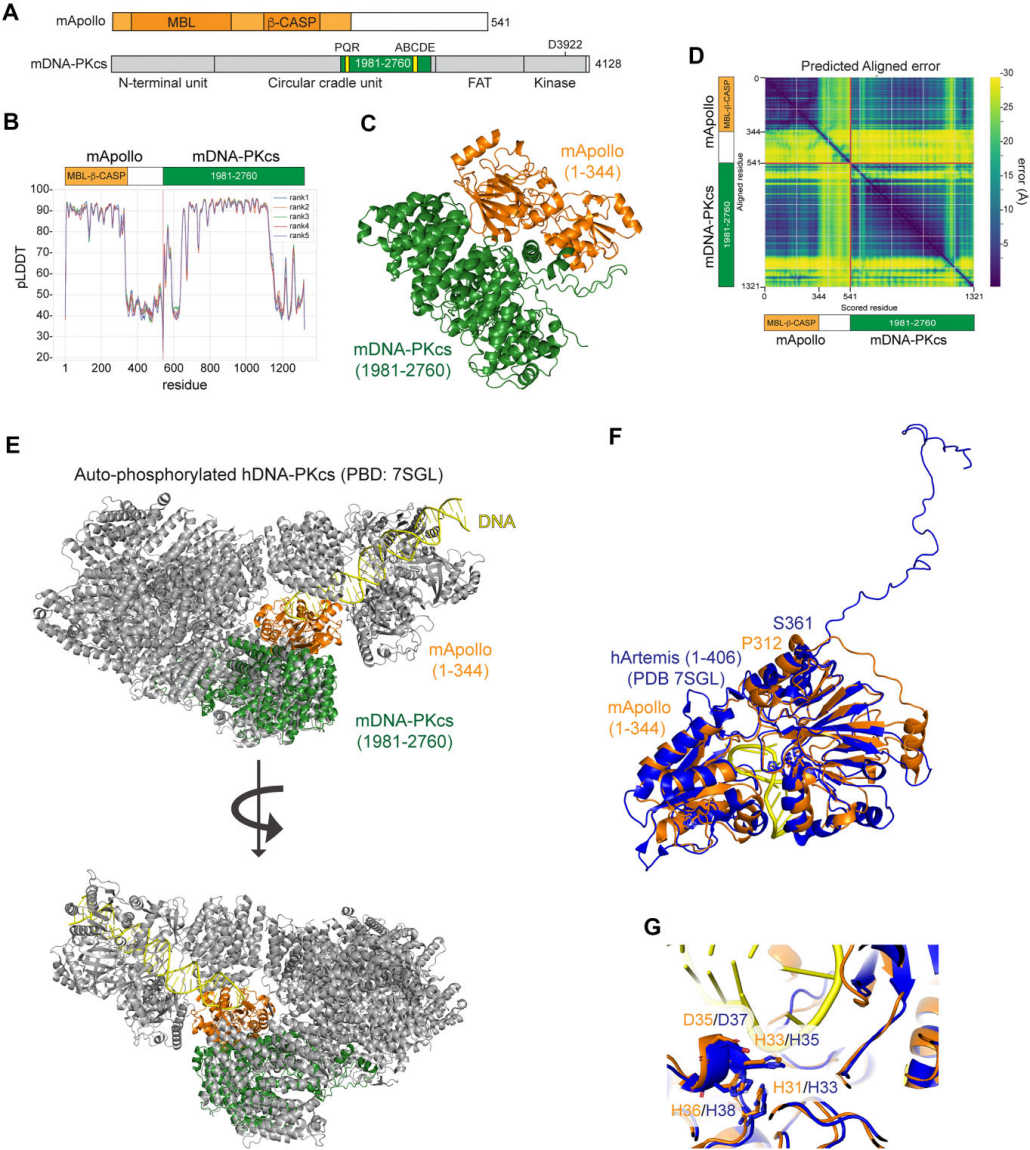
Phosphorylation on the DNA-PK ABCDE/Thr2609 cluster is predicted to allow Apollo access to telomere ends. (**A**) Schematics of mApollo and mDNA-PKcs. The metallo β-lactamase (MBL) and the β-CASP domains of Apollo and the N-terminal unit, the Circular cradle unit, the FAT and the Kinase domains of DNA-PKcs are indicated. Highlighted, the Apollo region (residues 1–344) predicted with high confidence by AlphaFold-Multimer modeling using AFsample and the DNA-PKcs region (residues 1981–2760) used in the modeling, with PQR and the ABCDE clusters. (**B**) The predicted Local Distance Difference Test (pLDDT) per residue position. (**C**) A model for the interaction of full-length mApollo and mDNA-PKcs (amino acids 1981–2760) as predicted by AFsample. Residues 345–541 in Apollo were removed from the model due to low confidence. (**D**) Predicted aligned error (PAE), as expected position error (*x,y*) at residue *x* if the predicted and true structures were aligned on residue *y*. (**E**) Superposition of (C) to the cryo-EM structure of autophosphorylated DNA-PK in complex with DNA (PBD: 7SGL) (7). (**F**,**G**) Comparison between the catalytic domains of hArtemis as in (PBD:7SGL) and mApollo model as superposed to PBD:7SGL (7) in (E), and enlargement on the catalytic residues. The key catalytic residues in Apollo and Artemis are indicated in orange and blue, respectively.

Autophosphorylation of the ABCDE/Thr2609 opens the DNA-PK groove and exposes the DNA end to Artemis(7). To investigate the possibility that phosphorylation of DNA-PK could similarly grant Apollo access to the ends, we used AFsample (69), an improved version of AlphaFold-Multimer (70) to query potential interactions between full-length Apollo and part of the circular cradle domain (residues 1981–2760) of DNA-PK. This part of DNA-PK contains the PQR/Ser2056 and ABCDE/Thr2609 clusters and interacts with Artemis after autophosphorylation (7) (Figure 5A). In total, 816 potential interactions were sampled, and the five highest ranking based on the reported ranking confidence were selected as a representative interaction mode (Supplementary Figure S7A). The N-terminal part (residues 1–344) of Apollo and residues 2140–2540 of DNA-PKcs were predicted with high confidence (pLLDT > 0.8) (Figure 5B–D), while the C-terminal half of Apollo was predicted with low confidence and was removed from further analysis. As expected, the predicted structure of Apollo overlapped substantially (RMSD: 0.692 Å) with the crystal structure of human Apollo (PDB: 5AHO) (80) with the exception of residues 314–344, which were not resolved in the crystal structure (Supplementary Figure S7B). Since these 30 residues are lacking in the crystal structure, it is likely that they are flexible and could potentially be interacting with other regions of DNA-PK not present in the model, as discussed below.

Importantly, the interaction between the catalytic domain of Apollo with DNA-PKcs was predicted with high confidence, indicated by the low predicted alignment error between Apollo and DNA-PKcs (Figure 5B–D). Superposition of the predicted model of DNA-PK with the cryo-EM maps of DNA-bound autophosphorylated DNA-PK (PBD: 7SGL) or unphosphorylated DNA-PK (PBD: 7SU3) (7) resulted in a similar RMSD of 3.057 and 3.386 Å (Figure 5E and Supplementary Figure S7C). However, the predicted model of the Apollo structure could be fitted only into the open cradle of autophosphorylated DNA-PK (PBD: 7SGL) (Figure 5E and Supplementary Figure S7C). Furthermore, in this latter conformation, the inferred interaction between the N-terminal of Apollo and DNA-PK allowed juxtaposition of the catalytic core of Apollo with the DNA end and of Apollo (residues 1–312) with Artemis (residues 1–361; PBD: 7SGL (7)), with a calculated RMSD of 2.304 Å and a near identical positioning of the catalytic residues (Figure 5F,G). These findings support the hypothesis that autophosphorylation of DNA-PK on the ABCDE/Thr2609 cluster promotes the conformational changes required to grant Apollo access to the telomere ends.

### Interaction between DNA-PKcs and the C-terminal region of Apollo is required for telomere protection

**Figure 6.**
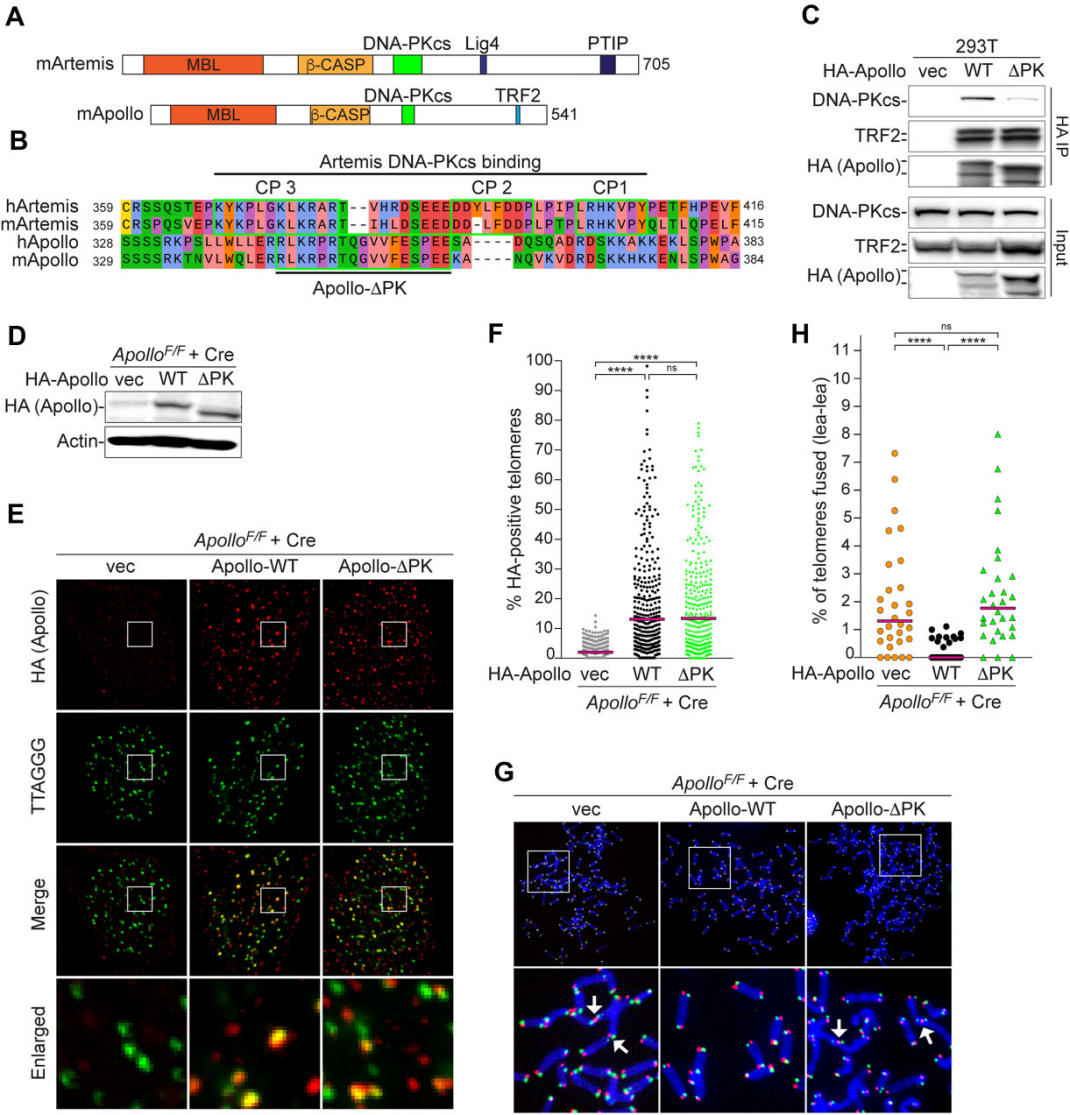
Apollo interaction with DNA-PK mediates telomere protection. (**A**) Schematics of mArtemis and mApollo. The MBL, the β-CASP, the known Lig4, PTIP (Pax transactivation-domain interacting protein) and TRF2-interacting domains, and the DNA-PKcs interacting regions are indicated. (**B**) MUSCLE alignment of human and mouse Artemis and Apollo C-terminal tails. Amino acids are colored according to physico-chemical properties (Zappo). Highlighted, the contact points (CP) of Artemis with DNA-PKcs (17) and the predicted DNA-PK-interacting region of Apollo, with a patch of positively charged amino acids followed by a patch of negatively charged ones. (**C**) Co-IP of endogenous DNA-PKcs and TRF2 with HA-mApollo-WT (WT) or HA-mApollo-ΔPK (ΔPK) in 293T cells. Pull-down was performed with anti-HA antibody. HA IP and input were analyzed by immunoblotting with anti-DNA-PKcs (top panels), TRF2 (middle panels) or HA (lower panel) antibodies. (**D**) Immunoblot of SV40LT-immortalized *Apollo^F/F^* MEFs transduced with either EV, HA-Apollo-WT or HA-Apollo-ΔPK 108 h after Hit & Run Cre-mediated deletion of endogenous Apollo. **(E** and **F)** IF-FISH analysis and quantification of Apollo localization at telomeres in the same MEFs as in (**D**). Graph represents 100 nuclei for each condition from *n* = 3 independent experiments (300 nuclei in total), with medians. **(G** and **H)** CO-FISH metaphase analysis and quantification of leading-end telomere fusions on MEFs treated as in (D). Graph represents 30 metaphases over *n* = 3 independent experiments with medians. Statistic by non-parametric Kruskal–Wallis ANOVA test for multiple comparisons (F, H). Statistical significance was indicated by ****P< 0.0001, ***P< 0.001, **P< 0.01, *P< 0.05; n.s., not significant.

In addition to the interaction between DNA-PK and Artemis discussed above, Artemis binds to DNA-PK through a short motif just C-terminal of the β-CASP (metallo‐β‐lactamase‐associated CPSF/Artemis/SNM1/PSO2) domain in a region of the protein that is largely disordered (7,16,17). The predicted structures of full-length Apollo did not show any similarity to Artemis beyond the β-CASP region (Figure 5F), and this region was not resolved in the X-ray structure of Apollo (80). However, our sequence alignments identified a short region in Apollo highly conserved in mammals with substantial similarity to the DNA-PK binding site in Artemis (Figure 6A,B and Supplementary Figure S8A,B). Indeed, endogenous DNA-PKcs was found to coimmunoprecipitate with HA-Apollo transfected into 293T cells, suggesting a direct interaction (Figure 6C). Importantly, a version of Apollo with a deletion of amino acids 344–360 (Apollo-ΔPK), removing the residues most similar to those of the Artemis DNA-PK interaction motif, had a reduced interaction with DNA-PKcs in 293T cells (Figure 6C).

We next examined the functional consequences of removing these residues from Apollo. Apollo-ΔPK interacted normally with TRF2 and localized to mouse telomeres at the same level as wild-type Apollo (Figure 6C–F). However, despite its localization to telomeres, Apollo-ΔPK failed to prevent telomere fusions when expressed in Apollo-deficient cells (Figure 6G,H). Although we cannot rule out that the deletion of amino acids 344–360 directly compromises Apollo’s catalytic activity, it is important to note that Apollo does not require its C-terminus, including the DNA-PKcs binding region, to process DNA ends *in vitro* (80,81). Therefore, these data suggest that direct interaction between DNA-PK and Apollo is required for the protection of leading-end telomeres.

## Discussion

**Figure 7.**
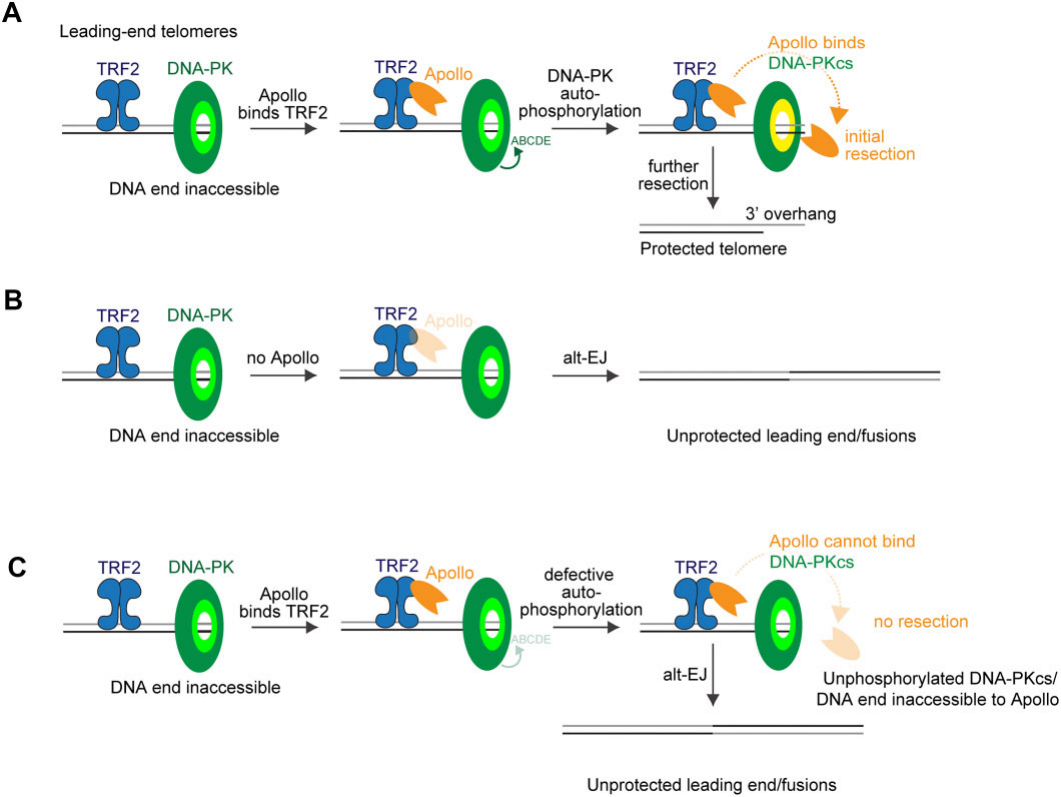
Proposed model for Apollo recruitment and access to leading-end telomeres after replication. (**A**) Apollo is recruited at telomeres by TRF2 binding. The physical presence of inactive DNA-PK at the blunt DNA end prevents any nucleolytic attack of the ends, including Apollo’s. After autophosphorylation of DNA-PKcs on the ABCDE/Thr2609 cluster and Apollo interaction with DNA-PKcs, Apollo gains access to the telomere ends and perform the initial resection required for the generation of the 3′overhang and for telomere protection. (**B**) In the absence of Apollo, when Apollo cannot be recruited by TRF2 or cannot interact with DNA-PKcs, the leading-end telomeres are not resected and are fused via alt-EJ. (**C**) In the absence of DNA-PK autophosphorylation, due to inhibition of the kinase activity of DNA-PK and/or to mutations affecting the kinase activity and/or the ABCDE/Thr2609 cluster of DNA-PK, Apollo cannot get access to the telomere ends, the leading-end telomeres are not resected and are fused via the alt-EJ.

Our data show that DNA-PK is required for the Apollo-dependent processing of leading-end telomeres, explaining the telomere fusion phenotype associated with DNA-PKcs inhibition and providing a rationale for the presence of DNA-PK at telomeres. Therefore, we propose a model (Figure 7) in which Apollo is recruited at telomeres by binding TRF2, while DNA-PK acts as gatekeeper preventing resection from Apollo or any other nuclease. The processing of the 3′-overhang is then initiated by DNA-PK autophosphorylation on the ABCDE/Thr2609 cluster, which grants Apollo exclusive access to telomeres (Figure 7A). When Apollo is absent (Figure 7B) or DNA-PK is inhibited/cannot be autophosphorylated (Figure 7C), the protective overhang is not formed, and the leading-end telomeres become vulnerable to alt-EJ.

The mechanism by which DNA-PK binds to and facilitates the nucleolytic attack by Apollo has striking similarities to how DNA-PK controls Artemis activity. Apollo seems to bind DNA-PK using a motif positioned just beyond the catalytic domain in the disordered C-terminal half, similar to Artemis. Furthermore, the (auto)phosphorylation of DNA-PKcs on the ABCDE/Thr2609 cluster is essential for DNA processing, while the S/T-Q sites of the nucleases themselves are not direct targets of DNA-PK kinase activity. However, some differences can also be noted. While DNA-PK is required for Artemis recruitment and opening of hairpins (20), Apollo recruitment to telomeres depends on TRF2 (Figure 3) (40,43,44,46,82). Furthermore, in the absence of DNA-PK, telomeres can be resected independently of Apollo (50), while hairpins can be opened only by Artemis once recruited by DNA-PK. Finally, in V(D)J recombination, DNA-PK kinase activity is not strictly required for hairpin opening by Artemis because it can be compensated for by ATM (20); in contrast, DNA-PK kinase activity is essential for resection at leading-end telomeres. These differences between Artemis and Apollo recruitment and activation could be explained by the biology of the telomeres themselves. Specifically, at telomeres, TRF2 recruits Apollo independently of DNA-PK while possibly having additional attributes that prevent ATM activation (38,50).

During DSB repair, DNA-PK autophosphorylation on the ABCDE/Thr2609 cluster occurs either in*cis*, when DNA-PK is monomeric and Artemis is granted access to the DNA ends (7), or in*trans*, during the transition from the XLF-mediated LR complex to SR (10). Due to the resemblance between Artemis and Apollo regulation, we suggest that, at newly replicated leading end telomeres, monomeric DNA-PK undergoes autophosphorylation in*cis*. Such a *cis-*acting mechanism avoids potentially dangerous synapsis of telomere ends. It is also possible that the autophosphorylation occurs in*trans*, if the DNA-PK complex is recruited on both the leading- and the lagging-strand telomere ends. After synapsis, the complex on the lagging end could then phosphorylate the one on the leading end and vice versa. In either case, DNA-PK must be activated without promoting c-NHEJ. How c-NHEJ is prevented is not known. In addition to DNA-PK acting as a monomer, fusion of telomeres might be prevented in a synaptic setting if the lagging-strand telomere has already been remodeled into the t-loop. It is also possible that the telomeric overhang of the lagging-strand prevents transition to the SR complex. Furthermore, although ATM deficient cells show no clear sign of telomere dysfunction, it is also possible that ATM might directly restrain telomere bound DNA-PK from synapsing and then promoting c-NHEJ, as seen at collapsed replication forks (28,30,83,84). Finally, TRF2 or one of its binding partners could prevent c-NHEJ independent of t-loop formation as it has been shown in several other settings (37,39,50).

Why DNA-PK is at telomeres when it is not strictly required for overhang formation (50,58–61) is not known. It has been suggested that the telomeric role of DNA-PK is the inhibition of alt-EJ (85), but data shown here and elsewhere (50,74) indicate that alt-EJ can also occur in the presence of DNA-PK. One possibility is that DNA-PK binds newly replicated leading-end telomeres after recognizing them as double-stranded DNA ends. Another possibility is that it is actively recruited to facilitate replication (86,87). In either case, DNA-PK blocks the processing of the blunt-ended telomeres until it autophosphorylates on the ABCDE/Thr2609 cluster and grants Apollo, and no other nucleases, access to the ends.

It will now be of interest to further investigate the interplay of DNA-PK and Apollo *in vitro* and compare it directly to the interplay between DNA-PK and Artemis.

## Supplementary Material

gkae105_Supplemental_File

## Data Availability

The computational model of DNA-PKcs/Apollo interaction has been submitted to ModelArchive under DOI 10.5452/ma-4yuqe. All raw images are available on FigShare at https://doi.org/10.6084/m9.figshare.25028588.
