# Supplementary material for: DNA-PK controls Apollo’s access to leading-end telomeres
Source: Nucleic Acids Res. 2024 Feb 26;52(8):4313–27. doi: 10.1093/nar/gkae105 (PMC11077071; doi:10.1093/nar/gkae105)
Supplement: gkae105_Supplemental_File [file gkae105_supplemental_file.pdf]

SUPPLEMENTARY DATA
SUPPLEMENTARY FIGURES

Figure S1

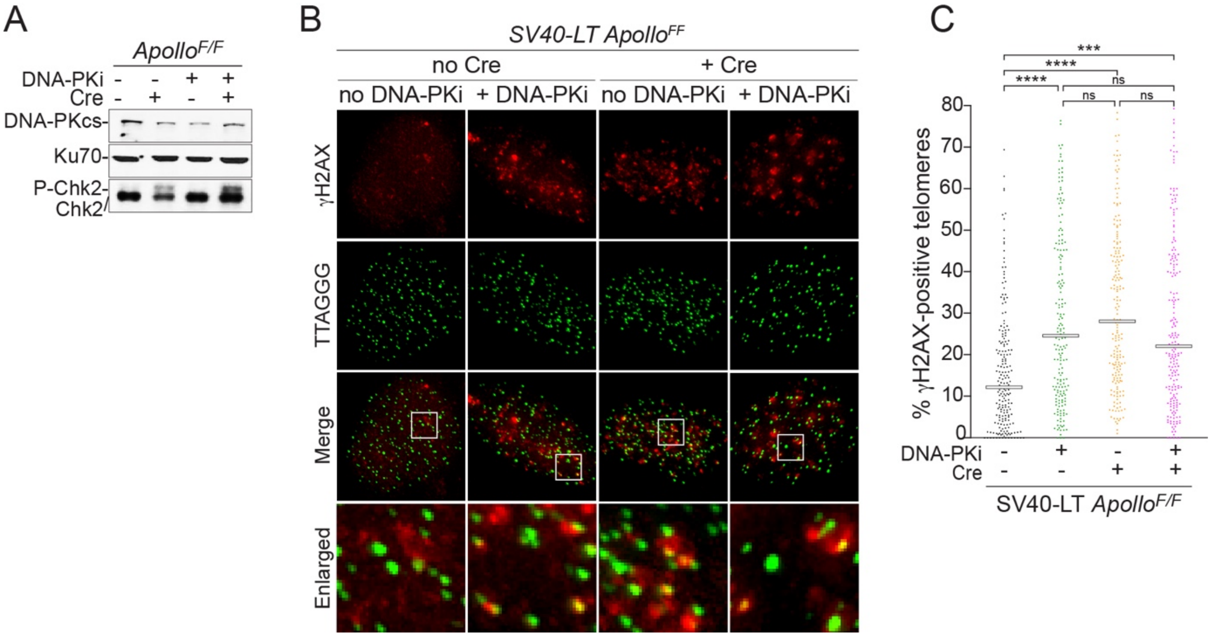

Supplementary Figure S1: DNA-PKi triggers Telomere Dysfunction Induced Foci (TIFs).
(A) Immunoblots for DNA-PKcs, Ku70, and phosphorylated Chk2 in SV40LT-immortalized Apollo$^{F/F}$ MEFs, 96 h after treatment with Hit & Run Cre and/or 24 h incubation with DNA-PKi.
(B) IF-FISH of Apollo$^{F/F}$ MEFs as in (A) 84 h after Hit&Run Cre infection and/or treatment with DNA-PKi for 24 h. TIFs are detected by immunofluorescence with antibodies for γ-H2AX (red) and the Telomeres-specific probe Alexa488-OO-(TTAGG)$_3$ (green).
(C) Quantification of the percentage of TIFs as in (B). Median bars from two independent experiments (100 nuclei per experiment per condition) is shown. Statistical analysis by non-non-parametric Kruskal-Wallis ANOVA test for multiple comparisons.

Figure S2

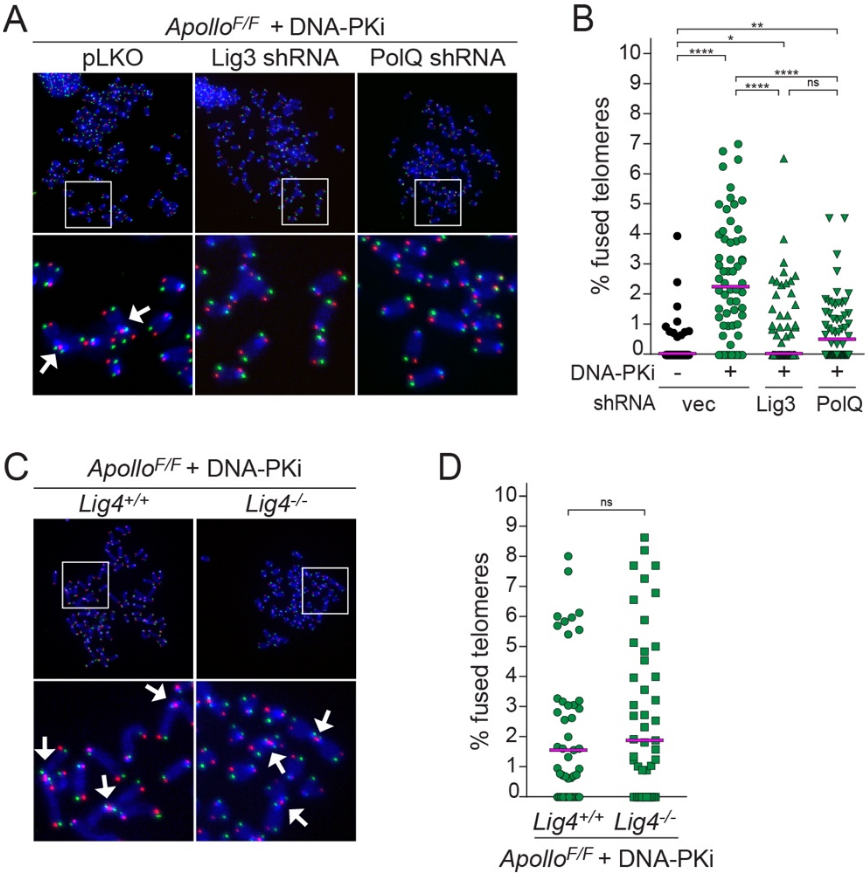

Supplementary Figure S2. Alt-EJ is responsible for leading-end telomere fusions in the absence of DNA-PKcs kinase activity.

(A) and (B) Representative metaphases and quantification with median bars of leading-end telomere fusions in 15 metaphases across four independent experiments (60 metaphases) for $Apollo^{F/F}$ MEFs infected with either an empty vector or shRNA against Lig3 or PolQ and treated with DNA-PKi for 24 h. White arrows highlight leading end telomere fusions.

(C) and (D) Representative metaphases and quantification of leading-end telomere fusions in SV40LT-immortalized $Apollo^{F/F}$ $Lig4^{+/+}$ and $Apollo^{F/F}$ $Lig4^{-/-}$ MEFs after 24 h treatment with DNA-PKi in 15 metaphases for three independent experiments (45 metaphases), with median bars.

Statistical analysis by non-parametric Kruskal-Wallis ANOVA test for multiple comparisons (B) or Mann-Whitney t-test (D).

Figure S3

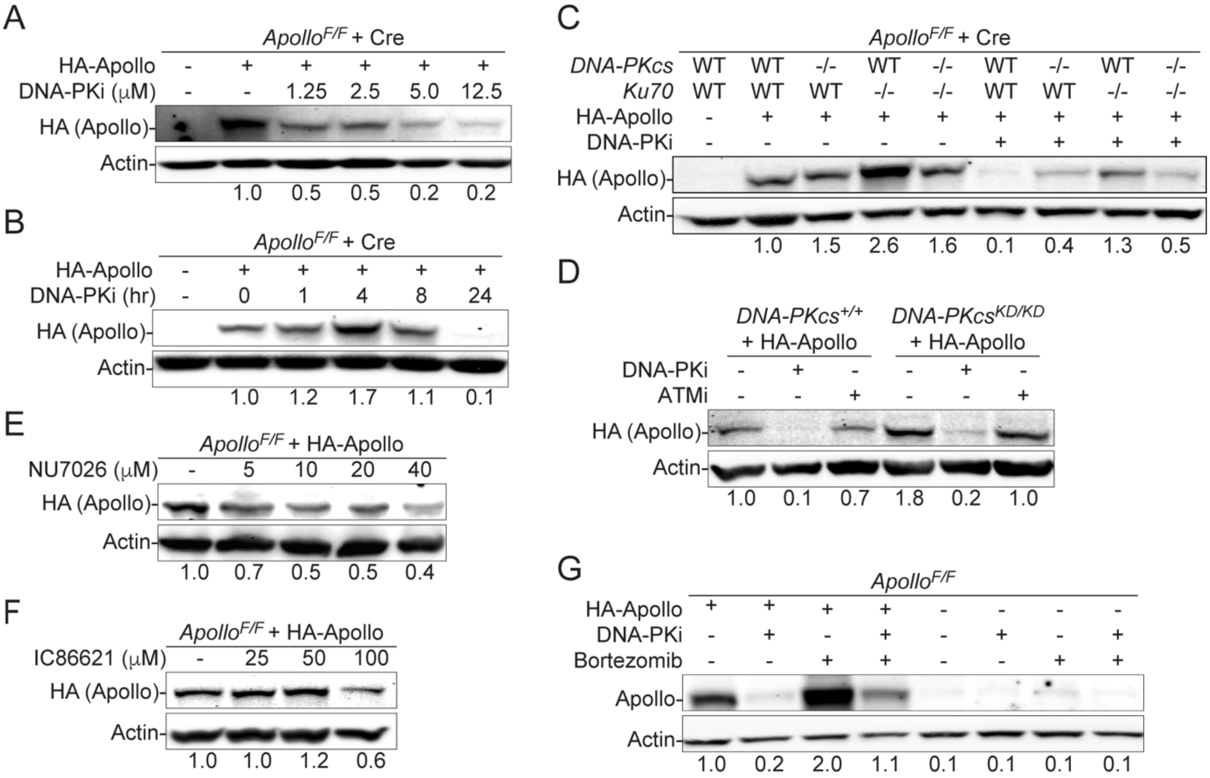

Supplementary Figure S3: DNA-PKi affects HA-Apollo stability independently from DNA-PKcs.
(A) and (B) Immunoblots for HA-Apollo and actin of Apollo$^{F/F}$ MEFs transduced with HA-Apollo or the empty vector after transduction with Cre and treatment with the indicated concentrations of DNA-PKi (NU7441) or with 12.5 μM of DNA-PKi (NU7441) for the indicated hours.
(C) Immunoblots for HA-Apollo and actin in SV40-LT-immortalized Apollo$^{F/F}$, Apollo$^{F/F}$ DNA-PKcs$^{-/-}$, Apollo$^{F/F}$ Ku70$^{-/-}$ or Apollo$^{F/F}$ Ku70$^{-/-}$ DNA-PKcs$^{-/-}$ MEFs transduced with an empty vector (EV) or HA-Apollo 120 h after Hit & Run Cre-mediated deletion of endogenous Apollo and/or 24 hr treatment with DNA-PKi (NU7441; 12.5 μM).
(D) Immunoblots for exogenous HA-Apollo and actin in SV40-LT-immortalized DNA-PKcs$^{+/+}$ and DNA-PKcs$^{KD/KD}$ MEFs transduced with HA-Apollo and left untreated or treated for 24 hr with DNA-PKi (NU7441; 12.5 μM) or ATMi (KU-55933; 2.5 μM).
(E) and (F) Immunoblots for HA-Apollo and actin in the same MEFs as in (A-B) after treatment with the indicated concentrations of IC86621 or NU7026.
(G) Immunoblots for Apollo (Atlas Antibodies) and actin of Apollo$^{F/F}$ MEFs transduced with HA-Apollo or the empty vector with or without treatment with DNA-PKi (NU7441; 12.5 μM) for 24 hrs and/or Bortezomib for 6 h.

Figure S4

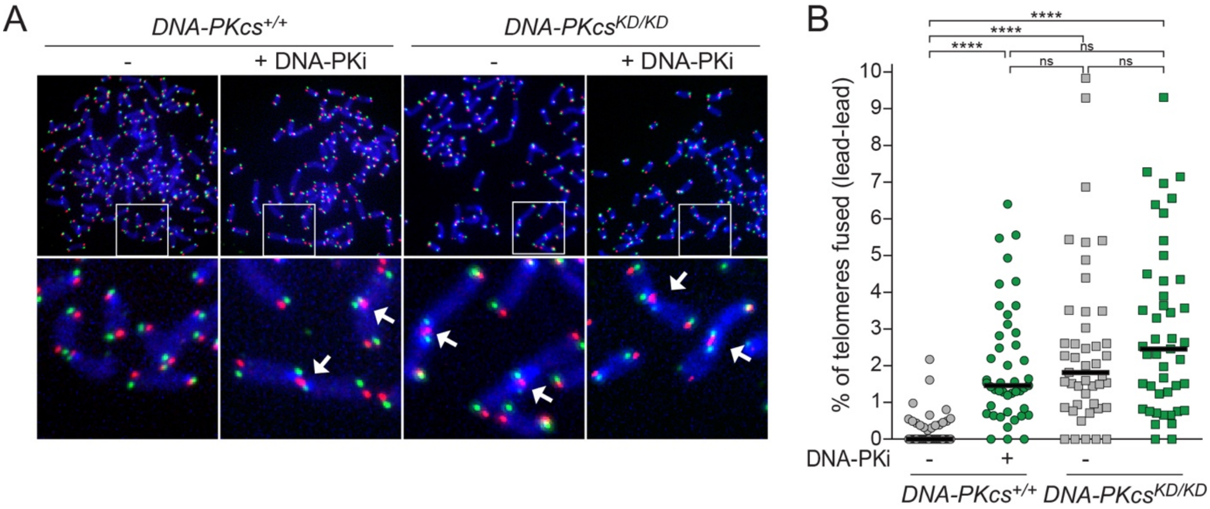

Supplementary Figure S4: DNA-PKi does not exacerbate the leading-end telomere fusions phenotype in DNA-PKcs$^{KD/KD}$ MEFs.

(A) and (B) CO-FISH metaphase analysis and quantification of leading-end telomere fusions in DNA-PKcs$^{+/+}$ or DNA-PKcs$^{KD/KD}$ MEFs with or without 24 h treatment with DNA-PKi. Leading and lagging-end telomeres were detected with Cy3-(TTAGGG)$_3$ (red) and Alexa488-(CCCTAA)$_3$ (green) probes, respectively. DNA was stained with DAPI (blue). Arrows indicate leading-end telomere fusions. Graph represents 45 metaphases over three independent experiments, with median.

Statistical analysis from non-parametric Kruskal-Wallis ANOVA test for multiple comparisons.

Figure S5

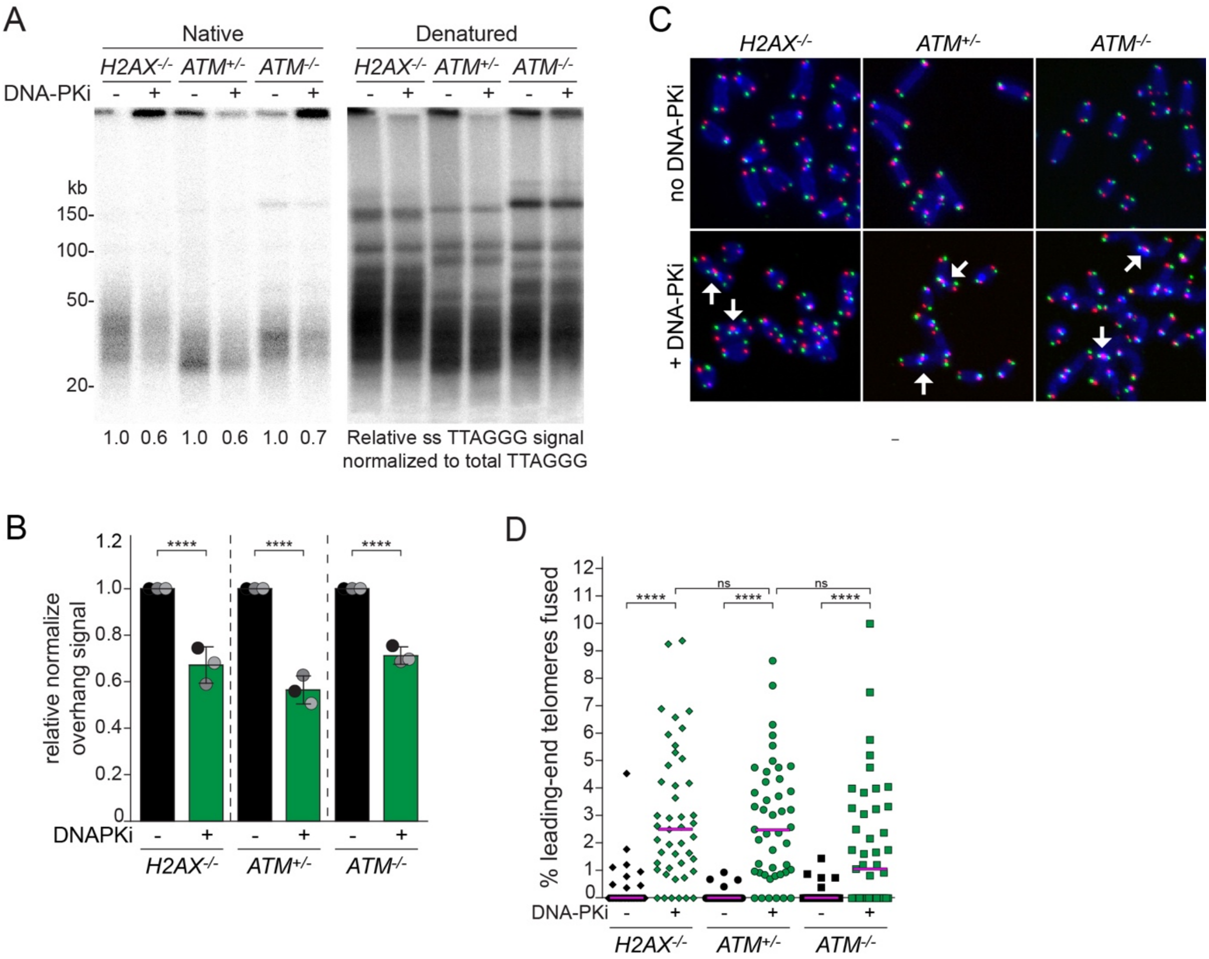

Supplementary Figure S5: DNA-PK kinase activity protects telomeres independently of H2AX or ATM.

(A) and (B) Telomeric overhang assay on SV40-LT-immortalized $H_2AX^{-/-}$, $ATM^{+/-}$ and $ATM^{-/-}$ MEFs (all $TRF2^{F/-}$) after 24 h incubation with DMSO or DNA-PKi, and quantification for three independent experiments. The normalized DMSO value of each cell line was set to 1, and all the other values for the same line were given relative to it, with means and SDs.

(C) and (D) CO-FISH metaphase analysis and quantification of leading-end telomere fusions on the same MEFs as in (J-K). The graph represents 15 metaphases for three independent experiments (45 total) with medians.

Statistical analysis by two-way ANOVA (B) Kruskal-Wallis ANOVA test for multiple comparisons (D).

Figure S6

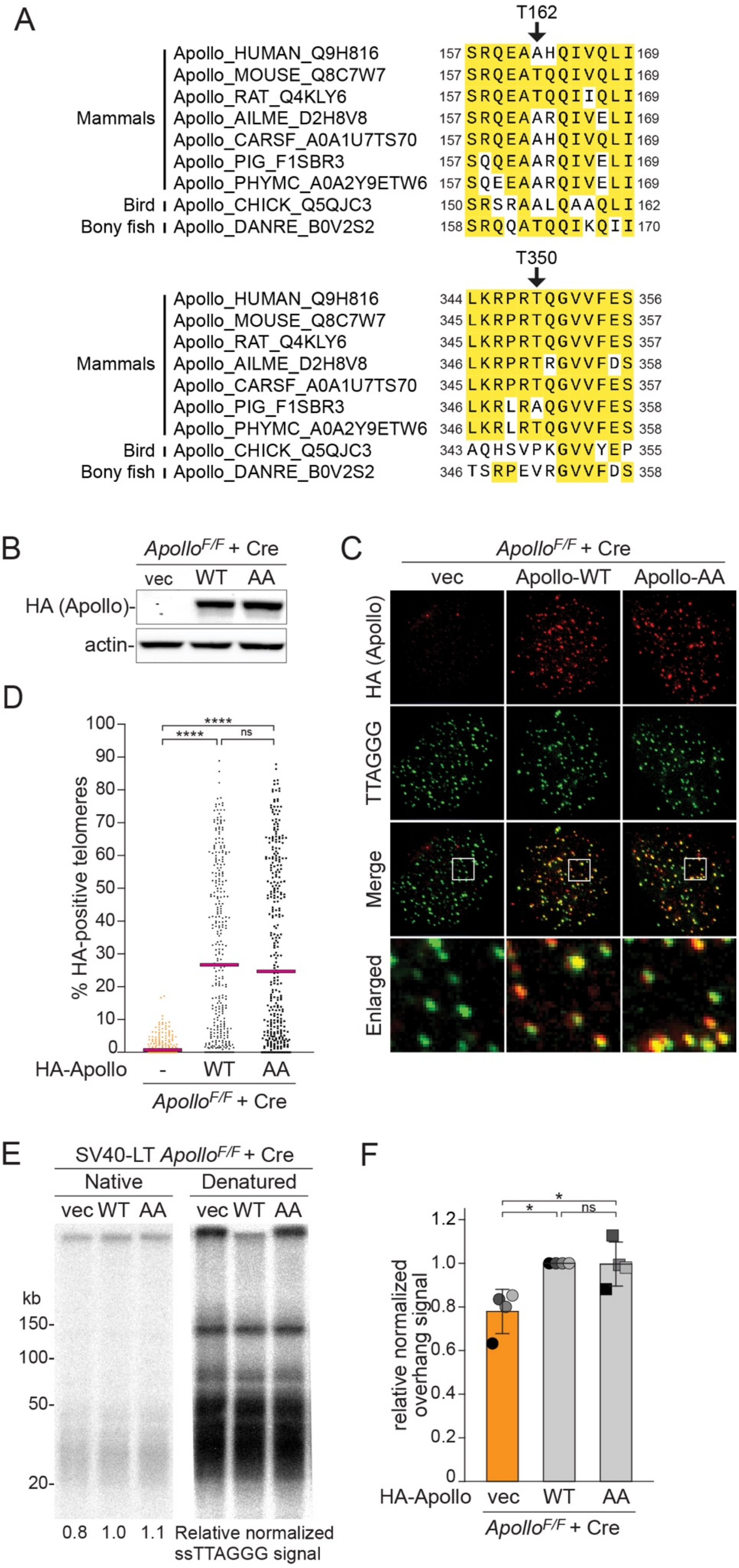
A
T162
Mammals
Bird
Bony fish
Apollo_HUMAN_Q9H816 157 SRQEAAHQIVQLI 169
Apollo_MOUSE_Q8C7W7 157 SRQEATQQIVQLI 169
Apollo_RAT_Q4KLY6 157 SRQEATQQIIQLI 169
Apollo_AILME_D2H8V8 157 SRQEAARQIVELI 169
Apollo_CARSF_A0A1U7TS70 157 SRQEAAHQIVQLI 169
Apollo_PIG_F1SBR3 157 SQQEAARQIVELI 169
Apollo_PHYMC_A0A2Y9ETW6 157 SQEEAARQIVELI 169
Apollo_CHICK_Q5QJC3 150 SRSRAALQAAQLI 162
Apollo_DANRE_B0V2S2 158 SRQQATQQIKQII 170
T350
Apollo_HUMAN_Q9H816 344 LKRPRTQGVVFES 356
Apollo_MOUSE_Q8C7W7 345 LKRPRTQGVVFES 357
Apollo_RAT_Q4KLY6 345 LKRPRTQGVVFES 357
Apollo_AILME_D2H8V8 346 LKRPRTRGVVFDS 358
Apollo_CARSF_A0A1U7TS70 345 LKRPRTQGVVFES 357
Apollo_PIG_F1SBR3 346 LKRLRAQGVVFES 358
Apollo_PHYMC_A0A2Y9ETW6 346 LKRLRTQGVVFES 358
Apollo_CHICK_Q5QJC3 343 AQHSVPKGVVYEP 355
Apollo_DANRE_B0V2S2 346 TSRPEVRGVVFDS 358
B
Apollo^F/F + Cre
vec WT AA
HA (Apollo)-
actin-
C
Apollo^F/F + Cre
vec Apollo-WT Apollo-AA
HA (Apollo)
TTAGGG
Merge
Enlarged
D
% HA-positive telomeres
100 90 80 70 60 50 40 30 20 10 0
****
****
ns
HA-Apollo - WT AA
Apollo^F/F + Cre
E
SV40-LT Apollo^F/F + Cre
Native Denatured
vec WT AA vec WT AA
kb
150-
100-
50-
20-
0.8 1.0 1.1 Relative normalized ssTTAGGG signal
F
relative normalized overhang signal
1.2 1.0 0.8 0.6 0.4 0.2 0
*
*
ns
HA-Apollo vec WT AA
Apollo^F/F + Cre

Supplementary Figure S6: Apollo S/T-Q sites phosphorylation is not required for telomere protection.

(A) Clustal Omega Alignment of Apollo in the indicated vertebrates. Only the amino acids around mouse T162 (top) and mouse T360 (bottom) are shown. In yellow, the residues matching the mouse sequence.

(B) Immunoblot for HA in SV40-LT-immortalized ApolloF/F MEFs transduced with either the empty vector (EV), HA-Apollo (WT), or HA-Apollo-AA (AA) 96 h after Hit & Run Cre-mediated deletion of endogenous Apollo. Actin is shown as loading control.

(C) and (D) IF-FISH analysis and quantification in the same MEFs as described in (B). 320 cells from four independent experiments were analyzed (80 nuclei/ experiment) for HA-Apollo and HA-Apollo-AA. For comparison, more than 160 nuclei from three independent experiments for pLPC are shown. Bars represent medians.

(E) and (F) Telomeric overhang assay and quantification from four independent experiments of MEFs as in (B). The normalized Cre value for HA-Apollo-WT was set to 1, and all the other values were given relative to it, with means and SDs.

Statistical analysis by non-parametric Kruskal-Wallis ANOVA test for multiple comparisons (B) and one-way ANOVA for multiple comparisons (D).

Figure S7

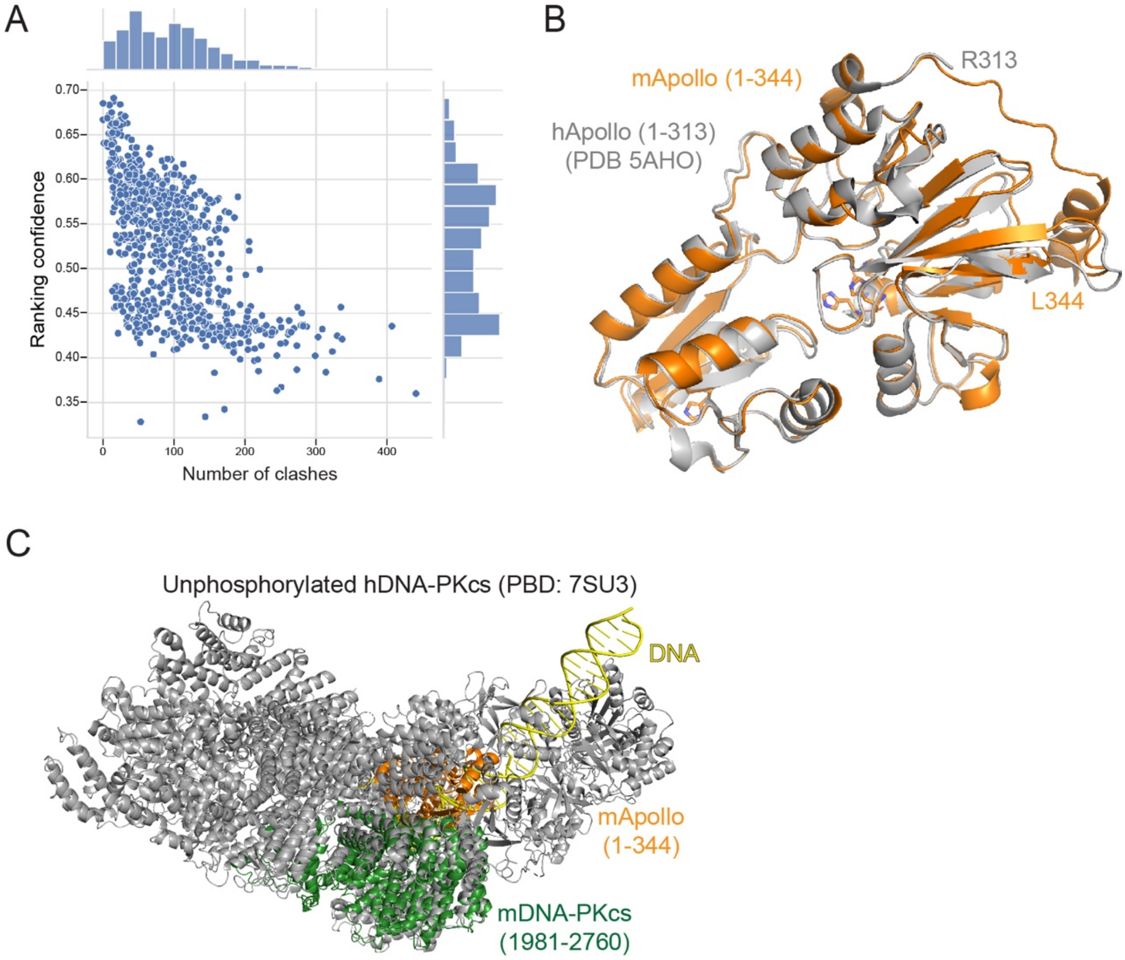

Supplementary Figure S7: Apollo interacts with DNA-PK.

(A) Ranking confidence for the 5,000 interaction models of mApollo and mDNA-PKcs (residues 1981-2760) generated by AFsample against the number of atomic clashes in the model. The model with the highest-ranking confidence and <20 clashes were energy minimized and chosen as the representative in (Figure 4).

(B) Superposition between the catalytic domains of mApollo as predicted by AFsample and hApollo as resolved in the crystal structure (PBD: 5AHO) (78). The last overlapping residues are indicated.

(C) Superposition of mAPollo and mDNA-PKcs as predicted by AFsample to the cryo-EM structure of inactive DNA-PKcs in complex with DNA (PBD: 7SU3) (7).

Figure S8

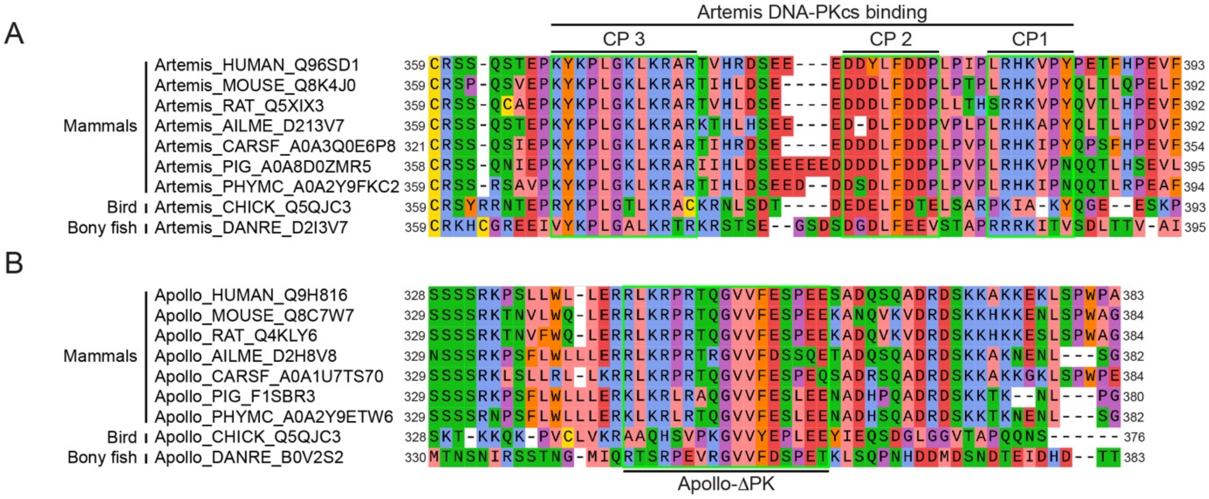

Supplementary Figure S8: Apollo S/T-Q sites phosphorylation is not required for telomere protection.

(A) Clustal Omega Alignment of Artemis C-tail in the indicated vertebrates. Amino acids are colored according to physico-chemical properties (Zappo). Highlighted, the contact points (CP) of Artemis with DNA-PKcs (17).

(B) Clustal Omega Alignment of Artemis C-tail as in (A). The predicted DNA-PK-interacting region of Apollo, with a patch of positively charged amino acids followed by a patch of negatively charged ones is highlighted.
